# Balancing Environmental Sustainability and Nutrition: Dietary Climate Impact in Relation to Micronutrient Intake and Status in a Swedish Cohort

**DOI:** 10.1016/j.cdnut.2025.107501

**Published:** 2025-07-05

**Authors:** Anna Stubbendorff, Ulrika Ericson, Ylva Bengtsson, Yan Borné, Emily Sonestedt, Elinor Hallström

**Affiliations:** 1 Nutritional Epidemiology, Department of Clinical Sciences Malmö, Lund University, Malmö, Sweden; 2 Diabetes and Cardiovascular Disease-Genetic Epidemiology, Department of Clinical Sciences Malmö, Lund University, Malmö, Sweden; 3 Division of Oncology, Department of Clinical Sciences Lund, Lund University, Lund, Sweden; 4 Department of Food and Meal Science, Faculty of Natural Science, Kristianstad University, Kristianstad, Sweden; 5 National Food Institute, Technical University of Denmark (DTU), Kongens Lyngby, Denmark; 6 Department of Agriculture and Food, Research Institutes of Sweden (RISE), Lund, Sweden

**Keywords:** sustainable diet, climate, micronutrient deficiency, nutrient adequacy, nutrient status

## Abstract

**Background:**

Dietary shift is necessary for improving public health, mitigating climate change, and achieving the Sustainable Development Goals. Adaption of climate-friendly diets might prevent chronic diseases and reduce mortality; however, consuming diets with a low climate impact have been suggested to increase risk of some micronutrient deficiencies.

**Objectives:**

This study aimed to examine whether self-reported dietary intake varying in climate impact is associated with nutritional risks and benefits based on both dietary intakes and blood concentrations of micronutrients.

**Methods:**

In the cross-sectional Malmö Diet and Cancer cohort (MDC, *n* = 25,970), dietary data were collected using a modified diet history method (food frequency questionnaire, diary, and interview). Blood samples were drawn and analyzed for micronutrients in different subgroups. Life cycle assessment data were used to estimate dietary greenhouse gas emissions (GHGE), from farm to fork. Quintiles of dietary climate impact were examined in relation to nutrient intake and status using linear and logistic regression.

**Results:**

The mean estimated dietary GHGE were 5.9 kg of carbon dioxide equivalents per day (females: 5.4, males: 6.6). Participants consuming a more climate-friendly diet had lower proportion of animal-sourced foods, energy intake, and intake of all micronutrients assessed (*n* = 17). Prevalence of anemia was higher in females consuming more climate-friendly diets, but the rates were low across all climate-diet groups (4.6% in Q1 compared with 3.3% in Q5; *P*-trend: 0.02), but not in males (*P*-trend: 0.131). No significant trends were observed in nutrient status of vitamin D, selenium, zinc, and folate across dietary GHGE quintiles.

**Conclusions:**

Despite a lower intake of micronutrients, more climate-friendly diets did not substantially increase risk of deficiencies. The study highlights the importance of measuring both nutrient intake and status when discussing nutritional consequences of sustainable eating.

## Introduction

Climate change is a growing threat to sustainability and human health. Food systems are key drivers of climate change, responsible for about one-third of global anthropogenic greenhouse gas emissions (GHGEs) [1,2]. Agriculture has a critical potential to mitigate climate change and might be one of the most cost-effective fields to approach [3]. Animal-based foods in general have higher climate impact than plant-based foods [4,5], and their consumption dominate dietary environmental impact in countries with high intake, such as Sweden [[6], [7], [8]]. A more plant-based diet could substantially reduce GHGE and other environmental impacts, thus increasing chances to keep environmental systems within the planetary boundaries [9,10]. Diets with lower environmental impact may also bring health benefits. On a population level, a shift toward climate-friendly diets, rich in plant-based foods has been estimated to reduce and prevent chronic diseases and mortality, particularly in high-income countries [[11], [12], [13]]. However, the health effects associated with climate-adapted diets depend on the dietary composition and quality [14]. Well composed climate-adapted diets, characterized as high in plant-based foods (e.g., vegetables, fruits, and whole grain products), moderate amounts of dairy, fish and poultry, and restricted intake of discretionary foods and meat [15] may improve diet quality in comparison with current western dietary patterns, for example, by lower intake of energy, total fat, saturated fat, sodium and higher intake of fiber, and whole grain [16]. However, climate-friendly diets may be associated with lower intake of micronutrients found in high quantities in animal-based foods (e.g., vitamin B-12, selenium, iron, zinc, calcium, and vitamin D) [[16], [17], [18], [19]]. Lower nutrient intake may especially be a concern among at-risk groups of the population with higher nutritional requirements, for example, children, adolescents, fertile and pregnant females, and older adults.

A challenge with studies assessing nutritional adequacy of diets is that they are often solely based on dietary intake, without accounting for bioavailability of nutrients and other factors affecting absorption and nutrient status. The importance of considering both nutrient intake and status when evaluating food sustainability has been raised due to the lower bioavailability of micronutrients in plant-based foods [20]. Bioavailability has been modeled on a dietary level [[21], [22], [23]] and assessed on a food item level [24], showing a substantial impact on nutrient absorption. Although measures of micronutrient intake together with nutrient status based on blood samples have been made to compare diets of, for example, omnivores, vegetarians, or vegans [[25], [26], [27], [28]], only limited studies [29] have, to our knowledge, combined assessments of environmental impact, nutrient intake from self-reported diets, and nutrient status.

The aim of our study was to compare nutritional adequacy of self-reported diets varying in climate impact, utilizing assessment of nutrient intake (including 17 nutrients) and nutrient status of vitamin D, selenium, zinc, folate, and hemoglobin derived from blood analyses in subgroups, previously analyzed. This study contributes to a broader dimension of measuring health impact of climate-friendly diets to advance the knowledge of nutritional risks and benefits of sustainable diets.

## Methods

### Malmö Diet and Cancer Study

The Malmö Diet and Cancer (MDC) Study is a prospective cohort study conducted in southern Sweden, with recruitment and baseline examinations between 1991 and 1996. The source population (*n* = 74,138 individuals) consisted of entire birth cohorts, all males aged 46–73 y and females aged 45–73 y living in Malmö at the time. Recruitment was carried out through community-directed (passive) invitations, that is, advertisements in public areas, and personal invitation letters (active recruitment). Exclusion criteria were limited Swedish proficiency or mental disability, hindering participants from filling out the baseline questionnaire (68,905 eligible individuals). A full description of the recruitment process for the MDC has been published elsewhere [30,31]. In total, 28,098 subjects completed the baseline examinations, corresponding to 41% of the eligible individuals. For this specific study, further exclusion was made for participants with incomplete information about food consumption (*n* = 2128), thus resulting in a total study population of 25,970 individuals (61% females) (Supplemental Figure 1). Ethical approval was given for all projects included in this article (LU 51–90, 567/2005, 652/2005, 23/2007, 2015/283). The participants provided written informed consent.

### Baseline examinations

Anthropometric measurements, blood pressure, and blood samples were assessed during the first visit [32]. A self-administered questionnaire was used at baseline to assess the participants’ demographic, lifestyle, socioeconomic, and social factors, current health, and medical history. Information was given about how to fill out a food diary and a food frequency questionnaire (FFQ). Around 2 wk later, during the second visit, a dietary interview was carried out, and the questionnaires were reviewed [30,32].

### Blood sample collection and laboratory analyses of nutrient status

Nonfasting blood samples (45 mL) were collected by trained nurses. Plasma and serum were separated within 1 h, and the samples were stored in a biobank at −80°C [31]. Serum values for hemoglobin (Hb), hematocrit, mean corpuscular volume, mean corpuscular hemoglobin, and mean corpuscular hemoglobin concentration from the cell count were analyzed directly. Subsequently, a series of nested case–control studies were conducted to analyze various nutrients in relation to prostate cancer in men and breast cancer in women. Serum and plasma from cases and controls were retrieved from the MDC biobank at different points of time.

Plasma folate concentration was analyzed in samples from women in 2007 (*n* = 1478) using a 2-step immunoassay with alkaline phosphatase, enzyme marking, and magnetic separation [33]. Serum vitamin D [serum 25-hydroxyvitamin D (25OHD), 25OHD_2_, and 25OHD_3_] was analyzed in samples from men and women in 2008 (*n* = 2903) with HPLC and parathyroid hormone (PTH) with the ImmuliteVR 2000 IntactPTH immunoassay (Diagnostic Products) [34]. 25OHD_2_ primarily comes from oral supplements, whereas 25OHD_3_ is produced endogenously and is present in some foods. In the samples analyzed, only 90 individuals had detectable concentrations of 25OHD_2_, among the majority of the population 25OHD_2_ concentrations were zero. Therefore, 25OHD_3_ was used in this study. Serum selenium and serum zinc in samples from women were analyzed in 2015 (*n* = 1943) by ALS Scandinavia AB in Sweden. Single-element standards, traceable to National Institute of Standards and Technology (NIST), were used for analyses on an ICP-SFMS (Thermo Element 2). A volume of 0.15 mL of serum was diluted to 10 mL with an alkaline solution containing 0.1% NH3 and 0.005% EDTA/Triton-X. The reference material, Seronorm, was sourced from Sero AS (Lot 0608414) and analyzed alongside the samples [35,36]. A flowchart with an overview of the different analyses can be found in Supplemental Figure 1.

### Dietary assessment and analyses of nutrient intake

Dietary intake was assessed during baseline examinations using a validated, modified diet history method consisting of 3 parts: *1*) a food diary for 7 consecutive days, for recording of intakes of prepared meals that vary from day to day (primarily lunch and dinner), cold beverages (including alcoholic beverages), and dietary supplements; *2*) a 168-item FFQ encompassing the preceding year, assessing consumption frequencies and portion sizes of food not covered by the food diary (breakfasts and snacks); and *3*) a 60-minute interview further assessing food choices, portion-sizes, and cooking methods. Each participant’s FFQ was checked for missing values and verified for no overlap between the registered intakes reported in the food diary as described in a previous publication [37]. Registered food intake was summarized from the food diary and the FFQ, and the mean intake of individual foods was expressed as grams per day. Calculations of energy and nutrient intake were conducted using a food composition database (PC-KOST2–93) from the Swedish Food Agency (1600 food items). Extreme values of major food groups, portion sizes, total energy, and nutrients were checked for errors. Calculated amounts of protein, fat, and carbohydrates were expressed as percentages of the nonalcoholic energy intake (E%). One retinol equivalent corresponds to 1 μg of dietary or supplemental preformed vitamin A (retinol), 2 μg of supplemental β-carotene, 6 μg of dietary β-carotene, or 12 μg of other dietary provitamin A carotenoids (such as α-carotene and β-cryptoxanthin) [15]. Niacin equivalents are calculated to account for both preformed niacin (vitamin B-3) from dietary sources and the niacin synthesized in the body from tryptophan, and 60 mg tryptophan is equivalent to 1 mg niacin equivalents [15]. Folic acid intake from supplements was multiplied by 1.7 to account for its higher bioavailability before being added to dietary folate intake.

The Medical Products Agency (Uppsala, Sweden) provided the names and ingredients of registered dietary supplements [38]. Manufacturers, sales agents, wholesalers, and merchants provided information on other dietary supplements. A modified diet history method similar to the method used in the MDC has previously shown good ranking validity and reproducibility compared with a reference method of 18-d weighed food records [39,40]. Compared with the reference method, the method in MDC yielded a higher absolute energy intake at group level of 18% [40]. The reported food intakes were higher than the reference method, with exception of fish, cream, and alcohol, as well as meat in females, and rice, pasta, and egg in males [39]. Energy-adjusted Pearson correlation coefficients between the methods for males and females were 0.75 and 0.75 for folate, 0.58 and 0.44 for zinc; and 0.46 and 0.44, for selenium, respectively [40].

Following a minor adjustment of the coding routines for dietary data in September 1994, which shortened the dietary interview from 60 to 45 min, dietary assessment version was introduced as a variable (old and new). The assessed energy intake was slightly lower after the adjustment but had no major impact on the ranking of the participants [37]. To control for seasonal variations, a variable with 4 categories corresponding to the season at baseline assessments was created. Participants were asked if they made any substantial dietary changes in the past due to ill-health or other reasons, and a binary variable was introduced. Potential dietary misreporters were defined as participants having a ratio of reported energy intake to basal metabolic rate outside the 95 % confidence interval of their calculated physical activity level [41]. Cases of diabetes, cancer, and carbon dioxide equivalent at baseline were identified through register linkage, with additional self-reported information for diabetes [42,43].

### Climate impact assessment

Dietary climate impact was estimated based on life cycle assessment (LCA) data, reported as GHGE per kilogram of edible food, compiled and harmonized by Hallström et al. [44]. LCA data used were chosen to be representative for the average Swedish consumption with regards to production method and origin. System boundaries include GHGE from cradle to consumer, including emissions from the primary production, energy use in processing, packaging, international transportation to Sweden, national transportation and distribution, and home transportation by the consumer, including losses and wastage of edible food along the food system studied. Emissions from home cooking were excluded because most food intake was expressed in raw weight. For a few food items, LCA data were complemented by data from other sources [7,45] which were harmonized to the same system boundaries as in Hallström et al. [44]. Reported food intake levels were matched with LCA data by adjusting for weight changes in cooking. For food items where reported intake of individual foods was grouped together (e.g., rice and pasta and fresh fruits), assumptions on consumed proportions were guided by consumptions levels in a Swedish dietary survey performed in 1997–1998 [46]. Composite dishes were divided into their respective food groups. The climate impact of spices, stock, and vinegar, representing small intake levels, were excluded due to data limitations. Climate impact was calculated for 117 subgroups of food items, using the available dietary intake data in the MDC. GHGE from dietary supplements was not included. Comparing GHGE of different food groups, all foods were assigned to one of the 12 food groups (Supplemental Table 1). The 12 groups were merged into 3 overall categories to compare the impact of plant-based, animal-based, and discretionary foods.

### Statistical analyses

Dietary climate impact was estimated as GHGE per day. To limit the influence of extreme values, the participants were divided into quintiles based on their calculated dietary GHGE (referred to as Q1–Q5). Quintiles were created sex-specific since males and females are known to emit different levels of GHGE due to differences in energy intake and dietary habits. Micronutrient intakes, and micronutrient status in relation to GHGE quintiles were analyzed using linear regression, and the estimated marginal means across quintiles were subsequently reported. Micronutrient intake was reported as dietary and as total intake (dietary + supplement) per day, and as dietary intake per 1000 kcal (nutrient density). Dietary micronutrient intake levels were compared with gender-specific and age specific (51–70 years) reference values for average requirement (AR) and recommended intake (RI) as outlined in the Nordic Nutrition Recommendations from 2023 [15]. Logistic regression with predicted margins was used for categorical variables. We used directed acyclic graphs to illustrate our interpretation of the relationship between the exposure and outcome variables (Supplemental Figure 2).The impact of adjusting for energy intake when examining the relationship between dietary GHGE and micronutrient intake has been explored in previous research [47]. In this study, we chose not to adjust for energy intake based on the following reasons: collinearity between dietary GHGE and energy intake, which would obscure the true relationship; RIs and ARs are based on absolute amounts; in studies of nutrient status, it is not common practice to adjust for energy intake; and climate goals are reported in absolute values. In the analyses of dietary nutrient intakes, we included the covariates age, season, and dietary assessment version. In the analyses with blood measures, we adjusted for age, season, and storage time of sample. Since analyses of Hb were carried out instantly, those analyses were adjusted only for season and age. We used reference values for Hb from the WHO [48]. Other reference values for serum/plasma concentrations were from the NIH [49]. Adjusted Pearson correlation analyses were used to calculate the correlation coefficient between nutrient intake and nutrient status in blood samples.

Statistical analyses were performed using IBM SPSS Statistics 27.0 and Stata SE18. A 2-sided *P* value of <0.05 was considered statistically significant.

### Sensitivity analyses

In sensitivity analyses, we assessed the associations between GHGE per 1000 kcal and nutrient intake per 1000 kcal. We modeled GHGE per day and GHGE per 1000 kcal, both as a continuous variable and as sex-specific quintiles. We further excluded potential misreporters, participants who reported dietary change in the past, participants with a history of cancer, diabetes, and cardiovascular disease (CVD) at baseline both individually and collectively, and premenopausal females. Additionally, we added alcohol consumption as a covariate for analyses of plasma folate. Lastly, we compared the associations between micronutrient status and different ways of modeling dietary GHGE, since it has been shown to alter the results for nutrient intake [47].

## Results

### Baseline characteristics and climate impact

Mean age was 58 y in females and 59 y in males (Table 1). Reported daily average energy intake was 2031 kcal for females and 2635 kcal for males, and mean BMI was 25.4 in females and 26.3 in males. Participants with higher dietary GHGE per day were more likely to have a higher energy intake, higher alcohol consumption, and a BMI of <25. They were also more likely to be younger, have a university degree, and be a smoker. The contribution (E%) of total and saturated fat and protein was slightly higher in the highest quintiles of dietary GHGE per day, whereas the contribution from carbohydrates was lower. Measured in grams per day, intake of protein was higher in diets with higher GHGE per day (Q1/Q5 females: 58 g/96 g; males: 72 g/122 g). For fiber, the total intake was higher in the highest quintiles of GHGE per day, whereas fiber per 1000 kcal was lower. In our sample, 4.4% had diabetes, 10.8% had a history of cancer, and 3.0% had a history of CVD. There was no substantial difference in dietary GHGE per day between these individuals and the rest of the population.

**TABLE 1:** Participant characteristics according to quintiles of dietary climate impact (greenhouse gas emissions per day) in 25,970 participants from the Malmö Diet and Cancer Study.

	Quintiles of dietary climate impact (kg CO_2_eq/d)^1^^,^^2^					
	1	2	3	4	5	All
Female (*n*)	3163	3163	3164	3163	3163	15,816
Age (y)	59.9 (8.1)	58.6 (8.2)	57.5 (7.9)	56.3 (7.7)	54.9 (7.0)	57.5 (8.0)
25–50 (%)	17.1	21.9	24.8	29.2	33.2	25.2
51–70 (%)	67.1	64.9	65.5	63.6	62.8	64.8
>70 (%)	15.8	13.2	9.6	7.2	4.0	10.0
BMI^3^	25.8 (4.4)	25.6 (4.3)	25.5 (4.2)	25.1 (4)	25.2 (4.3)	25.4 (4.2)
BMI above 25^3^ (%)	50.7	47.8	47.4	44.1	45.0	47.0
Current smokers^4^ (%)	25.4	26.3	27.7	28.5	33.1	28.2
High alcohol consumption^5^ (%)	0.4	1.0	1.8	2.4	6.4	2.4
High physical activity^6^ (%)	19.1	17.8	20.6	21.6	22.8	20.3
University degree^7^ (%)	9.4	12.4	14.4	17.6	21.6	15.0
Peripostmenopausal	98.8	98.7	98.8	98.4	98.6	98.6
Energy intake (kcal/d)	1578 (321)	1851 (322)	2013 (347)	2206 (393)	2506 (529)	2031 (501)
Kilocalories per kilogram of bodyweight	24.1 (6.7)	28.1 (7.1)	30.3 (7.3)	33.4 (8.2)	37.7 (10.8)	30.7 (9.4)
Fat (E%)	36.2 (6.2)	37.1 (6)	37.8 (5.8)	38.5 (5.8)	39.4 (5.9)	37.8 (6.1)
Saturated fat (E%)	15.3 (3.6)	16.0 (3.7)	16.4 (3.6)	16.9 (3.8)	17.5 (3.9)	16.4 (3.8)
Unsaturated fat (E%)	18.6 (3.4)	18.7 (3.2)	19.0 (3.1)	19.1 (3)	19.4 (3)	19.0 (3.2)
Protein (E%)	15.2 (2.6)	15.4 (2.4)	15.7 (2.4)	15.7 (2.4)	16.2 (2.5)	15.6 (2.5)
Carbohydrate (E%)	48.6 (6.3)	47.5 (6)	46.5 (5.7)	45.8 (5.7)	44.5 (5.8)	46.6 (6.0)
Dietary fiber (g)	16.2 (5.9)	17.8 (5.4)	18.6 (5.5)	20.1 (6.1)	21.6 (6.8)	18.9 (6.2)
Dietary fiber (g/1000 kcal)	10.5 (3.2)	9.9 (2.8)	9.6 (2.5)	9.5 (2.5)	9.0 (2.5)	9.7 (2.8)
kg CO_2_eq/d, mean (SD)	3.5 (0.5)	4.5 (0.2)	5.2 (0.2)	6.0 (0.3)	7.7 (1.2)	5.4 (1.5)
kg CO_2_eq/d (minimum–maximum)	1.2–4.1	4.1-4.8	4.8-5.6	5.6-6.5	6.5-22.8	1.2-22.8
kg CO_2_eq/1000 kcal	2.3 (0.5)	2.5 (0.4)	2.6 (0.5)	2.8 (0.5)	3.2 (0.7)	2.7 (0.6)
kg CO_2_eq/d/kg bodyweight	0.05 (0.01)	0.07 (0.01)	0.08 (0.01)	0.09 (0.01)	0.12 (0.03)	0.08 (0.03)
Male (*n*)	2030	2031	2031	2031	2031	10154
Age (y)	62.4 (6.9)	60.7 (7.1)	59.4 (7)	58.1 (6.7)	56.4 (6.3)	59.4 (7.1)
25–50 (%)	4.4	7.5	8.6	11.6	15.7	9.6
51–70 (%)	76.9	77.8	81.6	81.6	80.9	79.8
>70 (%)	18.7	14.6	9.8	6.8	3.3	10.6
BMI^3^	26.3 (3.5)	26.2 (3.4)	26.2 (3.5)	26.2 (3.4)	26.4 (3.7)	26.3 (3.5)
BMI above 25^3^ (%)	63.6	62.0	63.1	61.3	62.0	62.4
Current smokers^4^ (%)	23.3	26.7	27.0	31.0	35.6	28.7
High alcohol consumption^5^ (%)	1.6	3.6	6.6	8.9	16.5	7.4
High physical activity^6^ (%)	20.6	21.3	20.1	19.9	20.5	20.4
University degree^7^ (%)	10.5	11.8	13.6	15.0	15.3	13.2
Energy intake (kcal/d)	2039 (414)	2384 (410)	2616 (460)	2842 (538)	3294 (742)	2635 (677)
Kilocalories per kilogram of bodyweight	25.9 (6.8)	30.1 (7.1)	32.8 (7.9)	35.4 (8.8)	40.5 (11.4)	32.9 (9.9)
Fat (E%)	37.3 (6.3)	38.3 (6.1)	39.2 (6.1)	40.0 (6.2)	40.8 (6.1)	39.1 (6.3)
Saturated fat (E%)	15.3 (3.6)	16.1 (3.8)	16.7 (3.9)	17.3 (4.1)	17.8 (4.1)	16.7 (4.0)
Unsaturated fat (E%)	19.5 (3.6)	19.7 (3.3)	20.1 (3.3)	20.3 (3.4)	20.5 (3.2)	20.0 (3.4)
Protein (E%)	14.8 (2.4)	15.0 (2.3)	15.2 (2.3)	15.4 (2.4)	16.0 (2.6)	15.3 (2.4)
Carbohydrate (E%)	48.0 (6.4)	46.7 (6.1)	45.6 (6)	44.6 (6)	43.2 (5.9)	45.6 (6.3)
Dietary fiber (g)	18.4 (7.1)	20.3 (6.7)	21.5 (7.2)	22.3 (7.3)	24.4 (8.5)	21.4 (7.6)
Dietary fiber (g/1000 kcal)	9.3 (2.9)	8.9 (2.5)	8.6 (2.4)	8.3 (2.3)	7.9 (2.2)	8.6 (2.5)
kg CO_2_eq/d, mean (SD)	4.2 (0.6)	5.5 (0.3)	6.4 (0.3)	7.5 (0.4)	9.8 (1.7)	6.7 (2.1)
kg CO_2_eq/d (minimum–maximum)	0.8–5.0	5.0-5.9	5.9-6.9	6.9-8.2	8.2-24.2	0.8-24.2
kg CO_2_eq/1000 kcal	2.1 (0.4)	2.4 (0.4)	2.5 (0.5)	2.7 (0.5)	3.1 (0.7)	2.6 (0.6)
kg CO_2_eq/d/kg bodyweight	0.05 (0.01)	0.07 (0.01)	0.08 (0.01)	0.09 (0.01)	0.12 (0.03)	0.08 (0.03)

1 Quintiles of dietary greenhouse gas emissions per day for females/males—1: <4.1/<5.0; 2: 4.1–4.8/5.0–5.9; 3: 4.8–5.6/5.9–6.9; 4: 5.7–6.5/6.9–8.2, 5: >6.5/>8.2 kg CO_2_eq.

2 Values are means (SD) or percentages

3 Based on 25,929 participants (41 missing).

4 Based on 25,961 (9 missing).

5 Based on 25,949 participants (21 missing). High alcohol consumption defined as above 30 g/d for females and above 40 g/d for men.

6 Based on 25,838 participants (132 missing). Highest leisure time physical activity quintile.

7 Based on 25,904 (66 missing).

**FIGURE 1:**
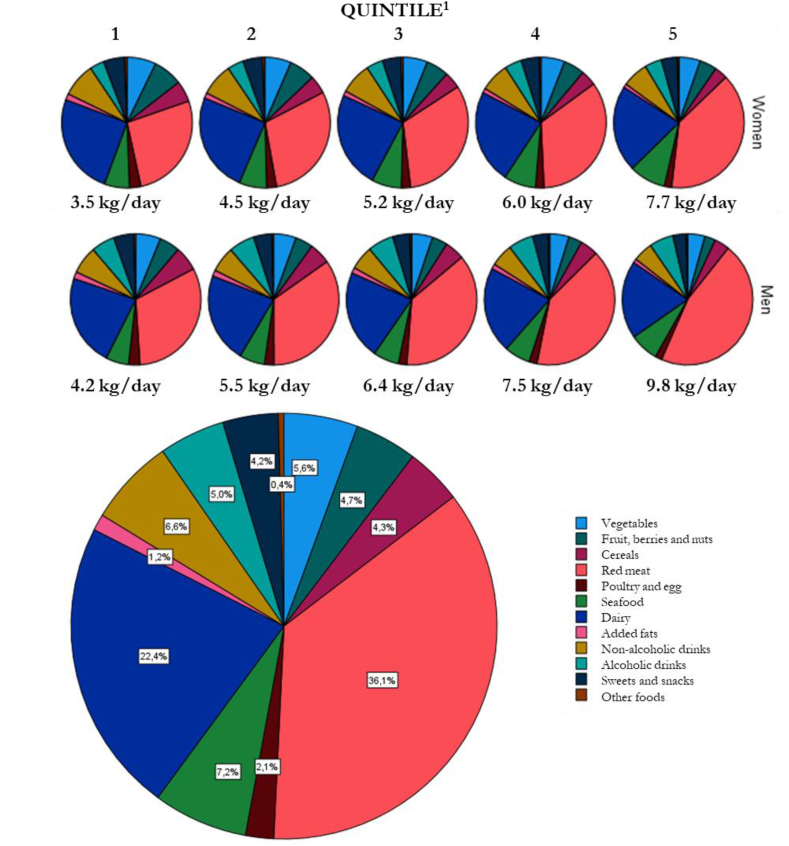
Contribution to dietary greenhouse gas emission (GHGE; kg CO_2_eq per day) from different food groups in 25,970 participants from the Malmö Diet and Cancer Study. ^1^Quintiles of dietary GHGE per day for females/males 1—<4.1/<5.0; 2: 4.1–4.8/5.0–5.9; 3: 4.8–5.6/5.9–6.9; 4: 5.7–6.5/6.9–8.2; 5: >6.5/>8.2 kg CO_2_eq/d.

The mean dietary GHGE in all participants of MDC were 5.9 kg of carbon dioxide equivalent (CO_2_eq) per participant and day, equivalent to 2.1 tons of CO_2_eq per year. Females had lower mean dietary GHGE of 5.4 kg CO_2_eq per day than males who had a mean dietary GHGE of 6.6 kg CO_2_eq per day (Table 1). Expressed as GHGE per 1000 kcal, the difference was nonsignificant between females and males. In the full sample, red meat was the food group contributing with most dietary GHGE, followed by dairy (Figure 1). In general, males had a higher proportion of GHGE from animal-based foods and lower from plant-based foods than females, although the difference was not significant. Animal-based, plant-based, and discretionary foods contributed to 62%–72%, 13%–20%, and 15%–18% of dietary climate impact, respectively, across quintiles of GHGE. The contribution of GHGE from red meat was higher in the higher quintiles (Figure 1, Supplemental Table 2). Dairy and fish also accounted for a higher proportion of the GHGE in the higher quintiles, whereas the contribution from vegetables and fruit was lower.

### Nutrient intake

Dietary GHGE was positively associated with all 17 micronutrients, as indicated in TABLE 2, TABLE 3 [15] (both when including and excluding intake from supplements). Consequently, a higher proportion of the subjects had a nutrient intake above AR and RI in the higher quintiles of dietary GHGE per day. During the 7-d dietary assessment period, 43% of females and 29% of males used some type of dietary supplements with ≥1 of the 17 studied micronutrients, and vitamin C was the most common supplement (Supplemental Table 3) [38].

**TABLE 2:** Micronutrient intakes across quintiles of dietary climate impact (greenhouse gas emissions per day) for 15,816 females from the Malmö Diet and Cancer Study^1^.

	Quintiles of dietary climate impact (kg CO_2_eq/d)^2^					β	*P*^3^
	1	2	3	4	5		
Vitamin A (RE^4^)							
Total intake	1571 (1329)	1830 (1321)	1947 (1320)	2106 (1317)	2342 (1329)	181.8	<0.001
Dietary intake	1360 (1102)	1606 (1095)	1715 (1095)	1861 (1093)	2063 (1102)	166.0	<0.001
Dietary intake above AR (530) (%)	94.9	98.8	99.6	99.5	99.8		
Dietary intake above RI (700) (%)	83.7	94.2	96.9	98.4	99.0		
Vitamin D (μg)							
Total intake	6.3 (4.1)	7.4 (4)	8 (4)	8.6 (4)	9.5 (4.1)	0.75	<0.001
Dietary intake	5.1 (2.5)	6 (2.5)	6.6 (2.5)	7.1 (2.5)	7.9 (2.5)	0.66	<0.001
Dietary intake above AR (7.5) (%)	12.9	21.5	29.5	38.2	50.5		
Dietary intake above RI (10) (%)	2.9	6.0	9.0	12.4	21.8		
Vitamin E (a-TE^5^)							
Total intake	12.5 (30.4)	13.4 (30.2)	14.8 (30.2)	16.2 (30.1)	16.8 (30.4)	1.14	<0.001
Dietary intake	7.8 (3.4)	8.8 (3.3)	9.4 (3.4)	10.4 (3.3)	11.5 (3.4)	0.91	<0.001
Dietary intake above AR (8) (%)	36.6	54.0	65.7	79.5	88.6		
Dietary intake above RI (9) (%)	24.1	36.9	47.4	62.3	75.9		
Thiamine (mg)							
Total intake	2.1 (7.6)	2 (7.5)	2.2 (7.5)	2.4 (7.5)	2.7 (7.6)	0.17	<0.001
Dietary intake	1 (0.3)	1.1 (0.3)	1.2 (0.3)	1.3 (0.3)	1.5 (0.3)	0.11	<0.001
Dietary intake above AR (0.07 MJ) (%)	99.9	100	100	99.9	99.8		
Dietary intake above RI (0.1 MJ) (%)	98.7	99.0	98.9	98.4	97.4		
Riboflavin (mg)							
Total intake	2.2 (5)	2.4 (4.9)	2.7 (4.9)	2.9 (4.9)	3.4 (5)	0.29	<0.001
Dietary intake	1.3 (0.5)	1.6 (0.5)	1.7 (0.5)	1.9 (0.5)	2.1 (0.5)	0.19	<0.001
Dietary intake above AR (1.3) (%)	48.6	75.7	83.9	91.2	95.7		
Dietary intake above RI (1.6) (%)	19.8	43.0	57.1	70.7	82.2		
Niacin (NE^6^)							
Total intake	30.8 (38.7)	35.2 (38.5)	38.7 (38.5)	41.9 (38.4)	48.8 (38.7)	4.3	<0.001
Dietary intake	24.6 (5.4)	28.8 (5.4)	31.6 (5.4)	34.5 (5.3)	40.1 (5.4)	3.66	<0.001
Dietary intake above AR (1.3/MJ) (%)	99.8	100	100	100	100		
Dietary intake above RI (1.6/MJ) (%)	99.3	100	100	100	100		
Vitamin B-6 (mg)							
Total intake	2.9 (17.2)	3.6 (17.1)	4.6 (17.1)	4 (17.1)	4.2 (17.2)	0.31	0.002
Dietary intake	1.4 (0.4)	1.7 (0.4)	1.8 (0.4)	2 (0.4)	2.2 (0.4)	0.18	<0.001
Dietary intake above AR (1.3) (%)	60.9	84.4	92.1	97.0	98.9		
Dietary intake above RI (1.6) (%)	28.2	50.8	66.8	80.5	92.2		
Folate (μg)							
Total intake	241 (315)	263 (313)	286 (313)	298 (312)	327 (315)	20.6	<0.001
Dietary intake	185 (66)	213 (66)	230 (66)	250 (65)	278 (66)	22.4	<0.001
Dietary intake above AR (250) (%)	10.8	22.2	31.1	44.2	59.7		
Dietary intake above RI (330^7^) (%)	2.1	3.6	5.8	10.5	22.2		
Vitamin B-12 (μg)							
Total intake	7.8 (148)	16.1 (147)	15.5 (147)	9.6 (147)	15.2 (148)	0.83	0.32
Dietary intake	4.5 (4.8)	5.5 (4.8)	5.9 (4.8)	6.5 (4.8)	7.6 (4.8)	0.73	<0.001
Dietary intake above AR (3.2) (%)	60.9	82.1	89.8	94.1	97.9		
Dietary intake above RI (4) (%)	39.7	61.3	73.9	81.0	91.6		
Vitamin C (mg)							
Total intake	152 (288)	164 (286)	186 (286)	199 (285)	217 (288)	16.5	<0.001
Dietary intake	84 (57)	102 (56)	111 (56)	119 (56)	134 (57)	11.6	<0.001
Dietary intake above AR (75) (%)	50.3	64.9	70.5	75.1	80.7		
Dietary intake above RI (95) (%)	33.7	48.3	55.7	60.3	68.2		
Calcium (mg)							
Total intake	853 (361)	1015 (359)	1107 (358)	1204 (358)	1364 (361)	121	<0.001
Dietary intake	830 (344)	992 (342)	1086 (341)	1183 (341)	1340 (344)	121	<0.001
Dietary intake above AR (750) (%)	59.3	80.4	86.5	90.9	93.6		
Dietary intake above RI (950) (%)	36.4	60.6	71.1	79.1	85.6		
Phosphorus (mg)							
Total intake	1083 (313)	1288 (311)	1412 (311)	1542 (310)	1762 (313)	161	<0.001
Dietary intake	1080 (312)	1285 (310)	1409 (310)	1539 (309)	1758 (312)	161	<0.001
Dietary intake above AR (420) (%)	99.8	100	100	100	100		
Dietary intake above RI (520) (%)	99.4	100	100	100	100		
Magnesium (mg)							
Total intake	264 (80)	301 (79)	322 (79)	351 (79)	391 (80)	30.3	<0.001
Dietary intake	255 (62)	292 (62)	314 (62)	341 (62)	381 (62)	30.2	<0.001
Dietary intake above AR (240) (%)	58.2	85.5	92.6	97.0	99.1		
Dietary intake above RI (300) (%)	17.8	39.9	58.7	73.7	87.7		
Potassium (g)							
Total intake	2.5 (0.6)	2.9 (0.6)	3.2 (0.6)	3.4 (0.6)	3.9 (0.6)	0.32	<0.001
Dietary intake	2.5 (0.6)	2.9 (0.6)	3.2 (0.7)	3.4 (0.6)	3.8 (0.6)	0.32	<0.001
Dietary intake above AR (2.8) (%)	27.2	55.8	72.2	83.9	92.8		
Dietary intake above RI (3.5) (%)	3.8	12.8	25.1	41.7	64.5		
Iron (mg)							
Total intake	13.6 (12.8)	15.3 (12.7)	16.6 (12.6)	17.9 (12.7)	19.8 (12.8)	1.51	<0.001
Dietary intake	10.7 (3.5)	12.3 (3.4)	13.4 (3.4)	14.6 (3.4)	16.6 (3.5)	1.14	<0.001
Dietary intake above AR (6) (%)	96.9	99.9	100	100	100		
Dietary intake above RI (8) (%)	83.8	96.1	98.3	99.7	99.8		
Zinc (mg)							
Total intake	9.2 (6)	10.9 (6)	11.9 (6)	13.2 (6)	15.1 (6)	1.41	<0.001
Dietary intake	7.4 (1.8)	8.9 (1.8)	9.8 (1.8)	11 (1.8)	13 (1.8)	1.32	<0.001
Dietary intake above AR (7.9) (%)	34.1	75.0	91.2	97.6	99.6		
Dietary intake above RI (9.5) (%)	8.3	29.9	55.8	79.9	95.7		
Selenium (μg)							
Total intake	33.2 (25.4)	39.1 (25.2)	43.3 (25.2)	46.8 (25.2)	52.5 (25.4)	4.62	<0.001
Dietary intake	26.3 (10.2)	31.1 (10.2)	34.3 (10.2)	37.4 (10.1)	43.2 (10.2)	4.02	<0.001
Dietary intake above AR (60) (%)	0.2	0.7	1.3	2.8	9.9		
Dietary intake above RI (75) (%)	0.0	0.1	0.1	0.4	2.1		

AR and RI are from Nordic Nutrition Recommendations [15]. Total intake is dietary intake + supplement intake. Recommendations for average requirement (AR) and recommended intake (RI) are from Nordic Nutrition Recommendations 2023.

1 Values are adjusted estimated means (SD) (based on general linear model) or percentages, adjusted for dietary assessment version, season, and age.

2 Quintiles of dietary greenhouse gas emissions per day for females 1: <4.1; 2: 4.1–4.8; 3: 4.8–5.6; 4: 5.5–6.5; 5: >6.5 kg CO_2_eq.

3 *P*-trend for general linear model.

4 Retinol equivalents.

5 α-Tocopherol equivalents.

6 Niacin equivalents.

7 Females of reproductive age are recommended to consume 400 μg/d (296 females had not undergone menopause).

**TABLE 3:** Micronutrient intakes across quintiles of dietary climate impact (greenhouse gas emissions/day) for 10,154 males from the Malmö Diet and Cancer Study^1^.

	Quintiles of dietary climate impact kg CO_2_eq/d (males)^2^					*β*	*P*^3^
	1	2	3	4	5		
Vitamin A (RE^4^)							
Total intake	1803 (1631)	2106 (1606)	2221 (1597)	2386 (1609)	2696 (1631)	246	<0.001
Dietary intake	1666 (1538)	1954 (1514)	2063 (1506)	2239 (1517)	2533 (1538)	201.9	<0.001
Dietary intake above AR (610) (%)	95.2	98.3	98.6	99.4	99.6		
Dietary intake above RI (800) (%)	85.4	93.6	95.1	97.7	97.7		
Vitamin D (μg)							
Total intake	7.8 (4.5)	8.9 (4.5)	9.7 (4.4)	10.2 (4.5)	11.5 (4.5)	0.87	<0.001
Dietary intake	7 (3.7)	8 (3.6)	8.8 (3.6)	9.4 (3.6)	10.5 (3.7)	0.87	<0.001
Dietary intake above AR (7.5) (%)	36.9	51.2	60.8	67.0	76.4		
Dietary intake above RI (10) (%)	13.6	23.8	30.0	37.9	50.2		
Vitamin E (a-TE^5^)							
Total intake	11.8 (22.8)	13 (22.5)	13.4 (22.3)	14.9 (22.5)	15.9 (22.8)	1.02	<0.001
Dietary intake	9.3 (4.7)	10.6 (4.6)	11.5 (4.6)	12.3 (4.6)	13.7 (4.7)	1.06	<0.001
Dietary intake above AR (9) (%)	43.0	63.3	73.7	79.4	88.5		
Dietary intake above RI (11) (%)	22.4	33.5	45.7	54.6	68.8		
Thiamine (mg)							
Total intake	1.6 (7.5)	2.3 (7.4)	2.2 (7.3)	2.5 (7.4)	3.1 (7.5)	0.3	<0.001
Dietary intake	1.2 (0.4)	1.4 (0.4)	1.5 (0.4)	1.7 (0.4)	1.9 (0.4)	0.15	<0.001
Dietary intake above AR (0.07 MJ) (%)	99.9	99.9	99.8	100.0	99.8		
Dietary intake above RI (0.1 MJ) (%)	97.6	98.2	97.5	97.4	96.3		
Riboflavin (mg)							
Total intake	2 (4)	2.4 (3.9)	2.6 (3.9)	2.9 (3.9)	3.4 (3.9)	0.32	<0.001
Dietary intake	1.6 (0.7)	1.9 (0.7)	2.1 (0.7)	2.3 (0.7)	2.6 (0.7)	0.24	<0.001
Dietary intake above AR (1.3) (%)	71.6	90.5	94.2	97.5	99.0		
Dietary intake above RI (1.6) (%)	44.1	70.6	78.9	86.5	93.9		
Niacin (NE^6^)							
Total intake	37.8 (102.1)	45.6 (100.6)	45.5 (100.1)	48.3 (100.8)	58.6 (102.2)	4.42	<0.001
Dietary intake	31 (7.6)	36.3 (7.5)	39.7 (7.5)	43.5 (7.5)	51.9 (7.6)	4.91	<0.001
Dietary intake above AR (1.3/MJ) (%)	100	100	99.2	100	100		
Dietary intake above RI (1.6/MJ) (%)	100	100	99.7	99.7	100		
Vitamin B-6 (mg)							
Total intake	2.4 (6.3)	2.7 (6.2)	2.9 (6.1)	3.1 (6.2)	3.7 (6.3)	0.31	<0.001
Dietary intake	1.7 (0.5)	2 (0.5)	2.2 (0.5)	2.4 (0.5)	2.7 (0.5)	0.24	<0.001
Dietary intake above AR (1.5) (%)	58.7	85.0	92.2	96.0	99.3		
Dietary intake above RI (1.8) (%)	39.7	67.1	79.5	88.8	97.3		
Folate (μg)							
Total intake	254 (334)	279 (329)	285 (328)	303 (330)	349 (334)	21.5	<0.001
Dietary intake	208 (79)	239 (78)	254 (78)	272 (78)	307 (79)	23	<0.001
Dietary intake above AR (250) (%)	22.8	37.8	47.8	57.2	72.3		
Dietary intake above RI (330) (%)	5.5	9.2	12.8	20.8	33.9		
Vitamin B-12 (μg)							
Total intake	9.4 (53)	9.8 (52)	11.4 (51)	11.1 (52)	13.5 (53)	0.95	0.011
Dietary intake	6 (6.7)	7 (6.6)	7.8 (6.6)	8.4 (6.6)	10 (6.7)	0.95	<0.001
Dietary intake above AR (3.2) (%)	80.6	93.0	96.9	98.1	99.4		
Dietary intake above RI (4.0) (%)	64.7	82.5	89.0	92.0	97.9		
Vitamin C (mg)							
Total intake	126 (243)	145 (239)	136 (238)	145 (239)	166 (243)	8	<0.001
Dietary intake	78 (59)	92 (58)	96 (58)	103 (58)	116 (59)	8.7	<0.001
Dietary intake above AR (90) (%)	31.7	41.4	45.0	50.2	56.5		
Dietary intake above RI (110) (%)	20.4	28.2	31.9	36.3	44.2		
Calcium (mg)							
Total intake	905 (438)	1073 (431)	1191 (429)	1298 (432)	1481 (438)	138	<0.001
Dietary intake	899 (434)	1067 (428)	1183 (425)	1291 (428)	1474 (434)	137	<0.001
Dietary intake above AR (750) (%)	65.3	83.1	87.3	91.6	94.3		
Dietary intake above RI (950) (%)	40.1	61.0	70.9	77.4	84.5		
Phosphorus (mg)							
Total intake	1308 (411)	1547 (404)	1701 (402)	1846 (405)	2150 (411)	198	<0.001
Dietary intake	1306 (409)	1544 (403)	1699 (401)	1845 (404)	2148 (409)	198	<0.001
Dietary intake above AR (420) (%)	99.1	100	100	100	100		
Dietary intake above RI (520) (%)	99.7	100	100	100	100		
Magnesium (mg)							
Total intake	309 (90)	356 (88)	385 (88)	412 (88)	472 (90)	38.1	<0.001
Dietary intake	306 (81)	352 (80)	380 (79)	407 (80)	466 (81)	37.6	<0.001
Dietary intake above AR (280) (%)	63.5	87.9	93.5	96.6	99.1		
Dietary intake above RI (350) (%)	22.3	48.4	66.3	75.3	90.7		
Potassium (g)							
Total intake	2.9 (0.8)	3.3 (0.8)	3.6 (0.8)	3.9 (0.8)	4.5 (0.8)	0.37	<0.001
Dietary intake	2.9 (0.8)	3.3 (0.8)	3.6 (0.8)	3.9 (0.8)	4.4 (0.8)	0.37	<0.001
Dietary intake above AR (2.8) (%)	52.3	80.6	87.1	93.6	98.2		
Dietary intake above RI (3.5) (%)	15.3	36.9	52.3	66.1	85.3		
Iron (mg)							
Total intake	17 (10.5)	18.4 (10.3)	19.5 (10.3)	20.9 (10.3)	24.1 (10.5)	1.67	<0.001
Dietary intake	14.6 (5.3)	16.6 (5.2)	17.8 (5.2)	19.4 (5.2)	22.6 (5.3)	1.87	<0.001
Dietary intake above AR (7) (%)	98.2	99.9	100	100	100		
Dietary intake above RI (9) (%)	92.9	98.8	99.5	99.8	100		
Zinc (mg)							
Total intake	10.5 (4.8)	12.5 (4.8)	13.7 (4.7)	15 (4.8)	18.1 (4.8)	1.78	<0.001
Dietary intake	9.3 (2.5)	11.2 (2.5)	12.5 (2.5)	13.8 (2.5)	16.9 (2.5)	1.80	<0.001
Dietary intake above AR (10.4) (%)	27.5	65.1	84.7	94.9	99.3		
Dietary intake above RI (12.4) (%)	6.7	24.4	50.2	71.6	93.5		
Selenium (μg)							
Total intake	37.7 (23.1)	43.1 (22.8)	46 (22.7)	49.4 (22.8)	57.2 (23.1)	4.53	<0.001
Dietary intake	32.3 (13.7)	37.5 (13.5)	40.7 (13.4)	44.4 (13.5)	52.4 (13.7)	4.72	<0.001
Dietary intake above AR (70) (%)	0.8	0.9	2.2	4.7	13.8		
Dietary intake above RI (90) (%)	0.3	0.2	0.4	0.5	3.3		

AR and RI are from Nordic Nutrition Recommendations [15]. Total intake is dietary intake + supplement intake. Recommendations for average requirement (AR) and recommended intake (RI) are from Nordic Nutrition Recommendations [15].

1 Values are adjusted estimated means (SD) (based on general linear model) or percentages, adjusted for dietary assessment version, season, and age.

2 Quintiles of dietary greenhouse gas emissions per day for males—1: <5.0; 2: 5.0–5.9; 3: 5.9–6.9; 4: 6.8–8.2; 5: >8.2 kg CO_2_eq.

3 *P*-trend for general linear model.

4 Retinol equivalents.

5 α-Tocopherol equivalents.

6 Niacin equivalents.

### Nutrient status

For vitamin D, selenium, zinc, and folate, there were no significant linear trends in mean serum/plasma concentrations across quintiles of GHGE per day and no difference in participants with status below reference concentrations. Hb was significantly higher in diets with higher dietary GHGE per day in both males and females (Table 4). The proportion of individuals with anemia (defined as Hb of <120 g/L for females and <130 g/L for males) was 4.0% in Q1 and 2.7% in Q5 (*P*-trend < 0.001) for the full sample. For females, the proportion with anemia was 4.6% in Q1 and 3.3% in Q5 (*P*-trend = 0.017), whereas for males, it was 2.7% in Q1 and 2.0% in Q5, but the difference was nonsignificant. The coefficient of variation for the different biomarkers were as follows: vitamin D, 31.7%; selenium, 18.3%; zinc, 17.8%; folate, 66.5%; and Hb, 8.5%.

**TABLE 4:** Micronutrient status for different subgroups from the Malmö Diet and Cancer Study, across quintiles of dietary climate impact (greenhouse gas emission/day).

	Quintiles of CO_2_eq/d^1^,^2^					β	*P*^3^
	1	2	3	4	5		
Females							
*n*	257	256	267	253	300		
Vitamin D (25OHD_3_ nmol/L)	88.1 (27.6)	89.6 (27.5)	88.2 (27.5)	91.4 (27.5)	85.9 (27.8)	−0.31	0.560
Below reference (50 nmol/L) (%)	5.3	6.2	5.8	4.4	7.7	0.06	0.441
*n*	356	377	371	405	434		
Serum selenium (ng/mL)	92.4 (16.7)	91 (16.6)	92.1 (16.6)	90.7 (16.6)	92.5 (16.7)	0.01	0.956
Below reference (63 ng/mL) (%)	1.0	3.7	1.4	3.2	2.9	0.14	0.178
*n*	356	377	371	405	434		
Serum zinc (μg/L)	10.6 (1.8)	10.3 (1.8)	10.2 (1.8)	10.4 (1.8)	10.3 (1.8)	−0.05	0.078
Below reference (10.6 μg/L) (%)	53.1	57.6	59.0	58.4	58.9	0.05	0.135
*n*	295	270	296	296	321		
Plasma folate (nmol/L)	12.7 (8.3)	12.1 (8.3)	13.1 (8.3)	12.4 (8.3)	12.1 (8.4)	−0.10	0.521
Below reference (6.8 nmol/L) (%)	22.6	24.7	16.9	14.2	23.3	−0.05	0.267
*n*	3155	3152	3155	3152	3147		
Hemoglobin (g/L)	135.8 (9.7)	136.1 (9.6)	136.1 (9.6)	136.1 (9.6)	136.6 (9.7)	0.15	0.007
Hemoglobin below reference (120) (%)	4.6	3.6	3.6	3.6	3.3	−0.07	0.017
HCT^4^	40.2 (3)	40.2 (2.9)	40.2 (2.9)	40.3 (2.9)	40.4 (3)	0.05	0.001
MCV^5^	89.1 (4.2)	89.2 (4.1)	89.2 (4.1)	89.4 (4.1)	89.6 (4.2)	0.12	<0.001
MCH^6^	30.2 (2.1)	30.1 (2.1)	30.1 (2.1)	30.2 (2.1)	30.3 (2.1)	0.03	0.020
MCHC^7^	338.9 (29.3)	337.9 (29.1)	338 (29)	337.8 (29.1)	338.2 (29.3)	−0.17	0.323
Males							
*n*	354	340	315	304	257		
Vitamin D (25OHD_3_ nmol/L)	85.2 (26)	89.2 (25.7)	86.5 (25.6)	83.8 (25.7)	88.2 (26.4)	0.02	0.966
Below reference (50 nmol/L) (%)	7.9	5.6	4.9	5.5	5.6	−0.09	0.289
*n*	2027	2031	2029	2030	2028		
Hemoglobin (g/L)	149.6 (10.3)	150.1 (10.2)	150.1 (10.2)	150.4 (10.2)	150.3 (10.3)	0.16	0.027
Hemoglobin below reference (120) (%)	2.7	2.0	1.9	2.0	2.0	−0.08	0.131
HCT^4^	43.8 (3.1)	43.9 (3)	43.9 (3)	44 (3)	44 (3.1)	0.05	0.036
MCV^5^	88.9 (4.2)	89 (4.1)	89 (4.1)	89.1 (4.1)	89.4 (4.2)	0.10	<0.001
MCH^6^	30.4 (4.4)	30.6 (4.3)	30.6 (4.3)	30.5 (4.3)	30.5 (4.4)	0.01	0.651
MCHC^7^	341.4 (12.2)	341.8 (12)	342.1 (12)	341.9 (12.1)	341.7 (12.2)	0.08	0.392

Model is adjusted for season, age, and storage time for vitamin D, selenium, zinc and folate. Hb and other values from cell count are adjusted for season and age. Serum status reference intervals are from Institutes of Health (NIH) and WHO (for hemoglobin).

HCT, hematocrit; MCH, mean corpuscular hemoglobin; MCV, mean corpuscular volume.

1 Values are adjusted estimated marginal means (SD).

2 Quintiles of dietary greenhouse gas emissions per day for females/males—1: <4.1/<5.0; 2: 4.1–4.8/5.0–5.9; 3: 4.8–5.6/5.9–6.9; 4: 5.7–6.5/6.9–8.2; 5: >6.5/>8.2 kg CO_2_eq.

3 *P*-trend for general linear model.

4 HCT, true relative percentage volume of erythrocytes (%).

5 MCV (fL): 50 L, 10^−15^ L.

6 MCH (pg; 10^−12^ g).

7 MCH concentration (g/L).

The correlations between total nutrient intake, including both diet and dietary supplements, and nutrient status were weak (*r* < 0.10), except for selenium (*r* = 0.31) and folate (*r* = 0.46) (Table 5). The correlations between total vitamin D and serum concentrations were *r* = 0.07 for females and *r* = 0.07 for males. When excluding dietary supplements, the correlations were stronger: *r* = 0.13 for females and *r* = 0.07 for males. Total selenium intake showed a significant positive correlation with serum selenium concentrations in females (*r* = 0.31), compared with selenium intake from food sources alone, which had a correlation of *r* = 0.11. The correlation between total zinc intake and serum zinc concentrations in females was negligible (*r* = 0.02). Dietary zinc intake from food alone showed a slightly negative correlation in females (*r* = −0.02), indicating minimal or no impact on serum zinc concentrations. There was a strong positive correlation between total folate intake and serum folate concentrations in females (*r* = 0.46). When considering folate intake from food sources alone, the correlation with serum folate concentrations remained positive (*r* = 0.15) but was less strong than total intake. There was a significant negative correlation between total iron intake and Hb concentrations in both females (*r* = -0.10) and males (*r* = -0.07). Excluding dietary supplements, the correlations with Hb concentrations were still negative in females (*r* = -0.05) and males (*r* = -0.04), suggesting that higher iron intake, even from food sources, was associated with lower Hb concentrations.

**TABLE 5:** Pearson correlation coefficients of total intake (dietary + supplement), dietary intake per 1000 kcal, and corresponding serum/plasma values, based on different groups with valid analyses from the Malmö Diet and Cancer Study.

Intake	Serum values						
	Females					Males	
	Vitamin D (*n* = 1333)	Selenium (*n* = 1943)	Zinc (*n* = 1943)	Folate (*n* = 1478)	Hb (*n* = 15,761)	Vitamin D (*n* = 1570)	Hb (*n* = 10,145)
Vitamin D, total	0.071∗∗					0.068∗∗	
Dietary vitamin D	0.132∗∗					0.073∗∗	
Dietary vitamin D/1000 kcal	0.141∗∗					0.109∗∗	
Selenium, total		0.306∗∗					
Dietary selenium		0.111∗∗					
Dietary selenium/1000 kcal		0.130∗∗					
Zinc, total			0.018				
Dietary zinc			−0.016				
Dietary zinc/1000 kcal			0.002				
Folate, total				0.456∗∗			
Dietary folate				0.148∗∗			
Dietary folate/1000 kcal				0.166∗∗			
Iron, total					−0.099∗∗		−0.073∗∗
Dietary iron					−0.050∗∗		−0.043∗∗
Dietary iron/1000 kcal					−0.018∗		−0.048∗∗

Analyzes are adjusted for age, season, dietary assessment method, and storage time of the blood samples (Hb was not adjusted for storage time since it was analyzed directly).

∗ Correlations significant at the 0.05 level.

∗∗ Correlations significant at the 0.01 level.

Characteristics and nutrient intake in the subgroups of participants with data on micronutrient status did not substantially differ from the entire population on which the analyses of nutrient intake is based (Supplemental Tables 4 and 5).

### Sensitivity analyses

When evaluating the associations between GHGE per 1000 kcal and nutrient intake per 1000 kcal, there was a significant positive linear trend for all 17 nutrients in females (Supplemental Tables 6 and 7). In males, there was a positive trend for 15 nutrients, but no differences were observed for vitamin E and iron. The way in which dietary GHGE was modeled as the exposure variable slightly influenced the associations with nutrient status (Supplemental Table 8). The quintile-based approach did not markedly change the significance or direction of associations compared with the absolute values. Using GHGE per 1000 kcal instead of GHGE per day generally showed stronger and more significant associations with blood parameters, particularly Hb and related measures, suggesting that GHGE per 1000 kcal might have a stronger association with micronutrient status. Nonetheless, the proportion of anemia remained statistically significant in all the different exposure models in females and remained nonsignificant in males and no other associations were altered. We observed similar patterns of associations between dietary intake and biomarkers when excluding misreporters, participants who reported recent dietary change, participants with prevalent disease (cancer, diabetes, and CVD or premenopausal females) (Supplemental Table 9). In the plasma folate analyses, we included alcohol consumption as an additional covariate, which did not significantly alter the observed associations (data not shown).

## Discussion

In this extensive Swedish cohort, individuals adhering to more climate-friendly diets demonstrated lower intake of animal-sourced foods, total energy, and lower intake of all 17 examined micronutrients. Despite lower absolute micronutrient intake, no differences were observed in biomarkers across GHGE quintiles. Anemia prevalence was higher in the diets with lower GHGE (4.0% in Q1 compared with 2.7% in Q5). However, the difference was only statistically significant in females. No significant differences were observed in nutrient status of vitamin D, selenium, zinc, and folate across quintiles of dietary GHGE per day.

### Nutrient intake

Diets with lower dietary climate impact had lower absolute intake of nutrients but higher nutrient density. Since recommendations on micronutrients are expressed as daily intakes, cutoffs for absolute intakes are of interest. We are aware of the concerns using absolute intakes without adjustment for energy due to potential misreporting. In our population, BMI was lower among those with high GHGE per day despite higher energy intake, indicating a potential systematic bias. Overweight individuals are often thought to underreport their dietary intake, although this phenomenon is not consistently observed across all studies [50]. Although adjusting for energy intake is a common practice in studies involving diets and nutrients, it is important to note that research focusing on self-selected dietary patterns (e.g., omnivores, vegetarians, and vegans) and micronutrient biomarkers typically does not include energy adjustments. Similarly, studies assessing the environmental impacts of diets and their alignment with climate goals generally do not use energy adjustments as a standard approach. In this article, we considered it relevant to relate GHGE to nutrient adequacy (AR and RI) using non–energy-adjusted models, as these analyses are only feasible without energy adjustments. Given the collinearity between dietary GHGE and energy intake, adjusting for energy would obscure the true relationship. While adjusting for total energy intake might eliminate the association between dietary climate impact and nutrient intake mediated by energy, it fails to account for the effects of overconsumption. Therefore, energy adjustment was not applied in the main analyses of this study. However, using both GHGE and nutrient intake per 1000, we observed similar associations as when we used the absolute amounts.

### Nutrient status

Despite the observed differences in nutrient intake between participants with varying dietary GHGE per day, surprisingly, only minor differences in nutrient status were observed. Factors such as nutrient absorption dynamics and nondietary influences could explain the weak to moderate correlations observed between nutrient intake and nutrient status. Including the dietary supplements in the correlations between nutrient intake and blood status generally increased the correlations; however, the opposite relation was unexpectedly observed for vitamin D. Correlations between nutrient intake and nutrient status were generally low.

Nutrient absorption is affected by micronutrient status; humans absorb many nutrients more efficiently when host status is low [[51], [52], [53]]. Overall food composition also influences nutrient absorption, impacting bioavailability in plant-based diets due to factors like phytic acid and the absence of the meat factor (which increases bioavailability of nonheme iron, the form of iron found in plant-based foods when consumed with meat, poultry, or fish). Absorption of dietary iron and zinc is inhibited by phytic acid, found in unrefined cereals, legumes, seeds, and nuts; therefore the bioavailability of iron and zinc is usually lower in plant-based diets including meat substitutes, due to a higher phytate content [54] and low or missing meat factor [55], and for iron due to its nonheme form. Intake of vitamin C or other acids are useful to enhance absorption of nonheme iron. Nutrient status may also be affected by other factors than food intake. For example, serum zinc is affected by stress, inflammation, infection, or albumin concentrations [56]. Serum zinc is also affected by food intake in general and might vary ≤20% during the day [57]. High BMI correlates with lower selenium concentrations [58]. Low concentrations of vitamin D have been associated with obesity, and being born outside Sweden [59], and status is affected by age and season due to sunlight exposure [60]. Other studies have reported a decline in Hb concentration with age [61], which we observed for males but not for females. Menopausal status is a critical determinant of iron requirements [15]. In our cohort, the majority (99%) were peripostmenopausal. To further explore this, we performed a sensitivity analysis restricted to premenopausal women. although the association between a climate-friendly diet and anemia was not statistically significant in this subgroup, the limited sample size likely restricted our ability to detect differences. Nonetheless, the lack of association may also reflect differing physiological demands and iron regulation across reproductive stages. These findings highlight the importance of life stage in nutritional epidemiology and suggest that menopausal status may influence the health implications of environmentally sustainable diets, particularly with regard to iron status.

In this study we adjusted for season and age in our analyses. The reason for excluding energy intake, BMI, and other covariates was that associations potentially mediated by energy intake and BMI also are of interest.

In this study, biomarkers of varying quality were used to assess nutrient status, revealing complexities in interpreting assessed blood concentration values for different nutrients. Serum 25-hydroxyvitamin D (25OHD) is currently considered the best marker of vitamin D status in humans [62]. Zinc concentrations in serum/plasma is widely used as biomarker of zinc status [56], even though serum/plasma zinc may be a better indicator of zinc status in extreme dietary conditions [63,64]. For accurate zinc status assessment, blood samples should ideally be taken fasting in the morning, as concentrations decrease throughout the day and after meals. In our sample, a substantial portion fell below the reference range, possibly due to nonfasting sample collection [65]. Serum selenium remains one of the most informative biomarkers for evaluating selenium status, despite this it has limitations as a marker for dietary intake [66,67]. The majority of the participants had a dietary intake of selenium below the RI levels, reflecting the cutoff used being higher than previous recommendations [68]. Although plasma folate is influenced by recent folate intake, it is considered to be a suitable marker of folate status in large epidemiological studies [69]. A combination of serum ferritin, transferrin, and Hb is recommended for determining iron status in humans [70]. In this study, only Hb and other values from the cell counting was available. The coefficients of variation were low for Hb, zinc, and selenium and high for vitamin D and folate in the studied populations.

### Results in relation to previous research

There is a lack of studies examining nutrient status in relation to dietary environmental impact. In a recent systematic review by Leonard et al. [18], only 1 RCT had micronutrient status as outcome; however, the exposure was based on percentage of animal-sourced protein and not climate impact per se. Other included studies were based solely on nutrient intake or were modeling studies. In a recent study of Swedish adolescents, the odds of iron deficiency were found to be 56% lower among girls with the highest than those with lowest dietary climate impact, whereas no similar associations were found for boys [29]. There are also several studies that have examined nutrient status among vegetarians or vegans compared with omnivores. Vegetarians have been shown to have a slightly lower intake of zinc and a lower zinc status than omnivores [26,27]. In a study, vegans had lower serum concentrations of vitamin D, iodine, and selenium, but a more favorable fatty acid profile than omnivores [25]. Another study found deficiencies of zinc among vegans, vitamin B-6 and niacin among vegetarians and of folic acid among omnivores [26]. A small German study, not taking supplements into account, showed a lower dietary intake of vitamin B-12 and higher intake of iron in vegans, but only minor differences in serum concentrations [27]. Many studies have reported that vegan diets contain higher iron intakes than omnivorous diets [[25], [26], [27], [28]], but findings related to anemia and iron deficiency have been inconsistent or contradictory [25,27,28,[71], [72], [73]]. In a study of Swedish adolescent females, iron deficiency was associated with restricted diets, such as being vegan, vegetarian, or avoiding red meat [73], but there was no difference in anemia prevalence. Another study examining nutrient status in relation to adherence to the EAT-Lancet diet, higher adherence was associated with an increased risk of anemia in women, a reduced risk of folate deficiency, and no significant differences in risk of deficiency for selenium, zinc, or vitamin D [74]. In our study, the participants were stratified according to their levels of GHGE and not meat consumption per se. This might explain why our results differ from studies on dietary patterns such as vegetarian and vegan. All measurements were taken at a single point in time, with participants reporting their habitual dietary intake over the past year. Blood analyses were performed to assess micronutrient status, aiming to reflect the long-term effects of dietary intake. Compared with modeling studies, this observational study captures the long-term effects of nutrient bioavailability and absorption, accounting for both inhibiting and promoting factors.

### Strengths and limitations

This study exhibits notable strengths, including the utilization of dietary intake data from a large population-based cohort, incorporating micronutrient status information in subgroups of the population. Furthermore, our implementation of a diet history method provides more detailed insights compared with a conventional FFQ. We observed similar patterns of associations when excluding misreporters, participants who reported recent dietary changes, and participants with prevalent disease, suggesting that the observed associations are robust and not significantly influenced by these specific subgroups. For the blood analyses the interassay coefficients of variation were low—for example, selenium: 3.4% [75] and zinc: 3.3% [36]—indicating a high reliability of our measurements. Nevertheless, certain limitations should be mentioned. A major limitation is the age of dietary data, which were collected in the 1990s and thus failed to capture current dietary habits. According to Swedish per capita consumption statistics, for example, the consumption of milk and yogurt has declined since 1990, whereas meat consumption has increased [76]. However, among adults in the age group represented in our study, we believe that dietary changes have not been substantial enough to undermine the relevance of our findings. Moreover, we believe that the dietary patterns captured in this cohort reflect broader nutritional transitions and may still offer insights that are applicable to other populations in Sweden and similar high-income countries. Our study included nutrient intake and nutrient status from a single time point, limiting the ability to assess long-term implications. Further, although nutrient intake data were available for the full sample, the biomarkers were only available for subgroups of the population. Misreporting of food intake may also compromise the accuracy of estimated dietary GHGE values and nutrient intake. Ranking participants assumes uniform misreporting and data deficiencies across all groups, an assumption that cannot be guaranteed. Nutrient status is affected by factors such as food matrix, nutrient interactions, and individual absorption rates, which may impact the accuracy of the intake-status relationship in our findings. Moreover, large variation in GHGE may exist also within individual foods depending on differences in local conditions and production systems. The absence of such detailed information introduces uncertainty in estimation of GHGE. Lack of information about food origin also introduces uncertainties for estimating micronutrient intakes, particularly for selenium, as soils selenium content varies significantly by region and directly affects the selenium content of food. The content of selenium in food depends heavily on the selenium concentration of the soil where the food originates from, and nutrient calculations might be a source of error. Soils in Europe have a low amount of selenium, leading to a low content in foods grown in the region [77], which might not be reflected in nutritional databases. In this study, only 0.13% (35 participants) of the population met the suggested climate target of 0.68 tons of GHGE per capita annually [7]. Attaining climate targets may necessitate more radical dietary changes, potentially impacting nutrient intake and status. Therefore, studying populations with lower dietary GHGE and assessing the nutritional effects of such diets through theoretical modeling becomes relevant for comprehensive insights. Lastly, to assess the wider environmental impact, other dimensions that GHGE are required.

Nonfasting blood samples collected at a single time point limits our ability to capture long-term nutrient status. For example, potential fluctuations in serum zinc concentrations over a 24-h period related to food intake have been observed [57]. Although correlations over time have been high for certain serum concentrations such as vitamin D [78], the complex relationship between dietary intake and serum concentrations, influenced by various factors, remains a challenge. Notably, the study lacks analyses of crucial serum values such as vitamin B-12 and ferritin [79], relying solely on Hb for iron status screening. Alterations in ferritin concentrations can occur before changes in Hb status [73], making ferritin the preferred marker for assessment. Despite efforts to mitigate the impact of measurement errors by checking all food diaries and FFQs during the dietary interview in the data collection phase, measurement error in the dietary data cannot be entirely avoided. The combination of dietary assessment methods used in MDC resulted in dietary data of high relative validity (energy-adjusted correlation coefficients between 0.28 and 0.77 for micronutrients), but it may not eliminate all sources of measurement uncertainty [40].

### Future studies

Future research should aim to include a broad spectrum of populations, incorporating diverse demographics such as those in low-income or middle-income countries and younger age groups, for example, children and adolescents. Analyzing current dietary patterns and the impact of newer food options could provide valuable insights. The landscape of available food options has evolved since the data collection period for this cohort, witnessing increased availability of meat substitutes alongside a growing prevalence of highly processed foods. Fortification and, consequently, the intake of micronutrients might differ when comparing different periods. Future studies should take into account that sustainable diets are a wide concept including a broad range of aspects [80]. For a more holistic sustainability perspective complementary assessments of additional perspectives beyond climate impact and nutrient adequacy are required. Lastly, when studying nutrient adequacy of diets, comprehensive assessments of all relevant nutrients and more biomarkers should be included to enhance reliability.

### Conclusions

We found that although diets with a lower climate impact were linked to lower micronutrient intake, there were no significant associations with micronutrient status of vitamin D, selenium, zinc, and folate. Although anemia was more prevalent in climate-friendly diets, the magnitude of the difference was small. Ultimately, more climate-friendly diets were associated with lower micronutrient intake, but no substantial increased risk of deficiency. This study emphasizes the importance of evaluating both nutrient intake and status when considering nutritional consequences of sustainable diets, contributing to a broader understanding of the health impact of climate-friendly diets.

## Author contributions

The authors’ responsibilities were as follows – AS, ES, EH, UE: designed the research; AS: conducted the analyses and made the conceptualization and visualization; AS, EH: wrote the original draft of the manuscript; UE, ES, EH, YB, YB: contributed to the interpretation of results and revision of the manuscript; EH: had primary responsibility for final content; and all authors: have read and approved the final version.

## Data availability

Data described in the manuscript can be made available upon request pending application and approval by the chair of the steering committee for the cohort.

## Funding

This study was supported by the Swedish Heart-Lung foundation (grant 20200482), Crafoord foundation (grant 20210674), and Agenda 2030 Graduate School, Lund University. The funding agencies had no influence on the design, analyses, or writing of this paper.

## Conflict of interest

YB reported an agreement on receiving honoraria for lectures from AstraZeneca. All other authors report no conflicts of interest.
