# Supplementary material for: Balancing Environmental Sustainability and Nutrition: Dietary Climate Impact in Relation to Micronutrient Intake and Status in a Swedish Cohort
Source: Curr Dev Nutr. 2025 Jul 5;9(8):107501. doi: 10.1016/j.cdnut.2025.107501 (PMC12355999; doi:10.1016/j.cdnut.2025.107501)
Supplement: multimedia component 1 [file mmc1.docx]

**Online supplementary material**

Supplement to: Stubbendorff A, et al. Balancing environmental Sustainability and Nutrition: Dietary Climate Impact in Relation to Micronutrient Intake and Status in a Swedish Cohort.

Supplemental figure 1. Flow chart of participants from the Malmö Diet and Cancer Study included in different analyses in this paper.


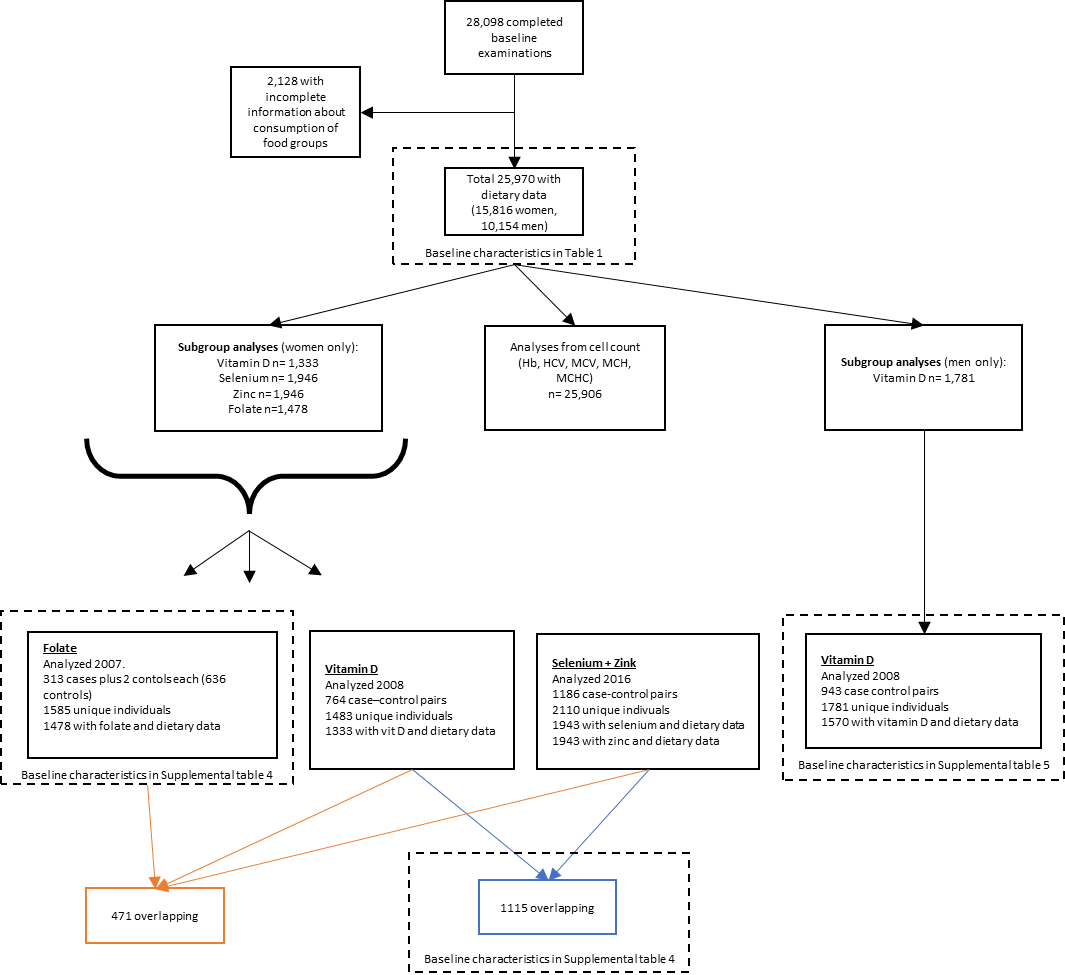


**Supplemental figure 2.** Directed Acyclic Graphs (DAGs) displaying assumptions about the relationship between variables in this project. The red circles are covariates to adjust for in the models. Models of nutrient intake are adjusted for age, dietary assessment method and season. Models of nutrient status is adjusted for season, age and storage time of sample.


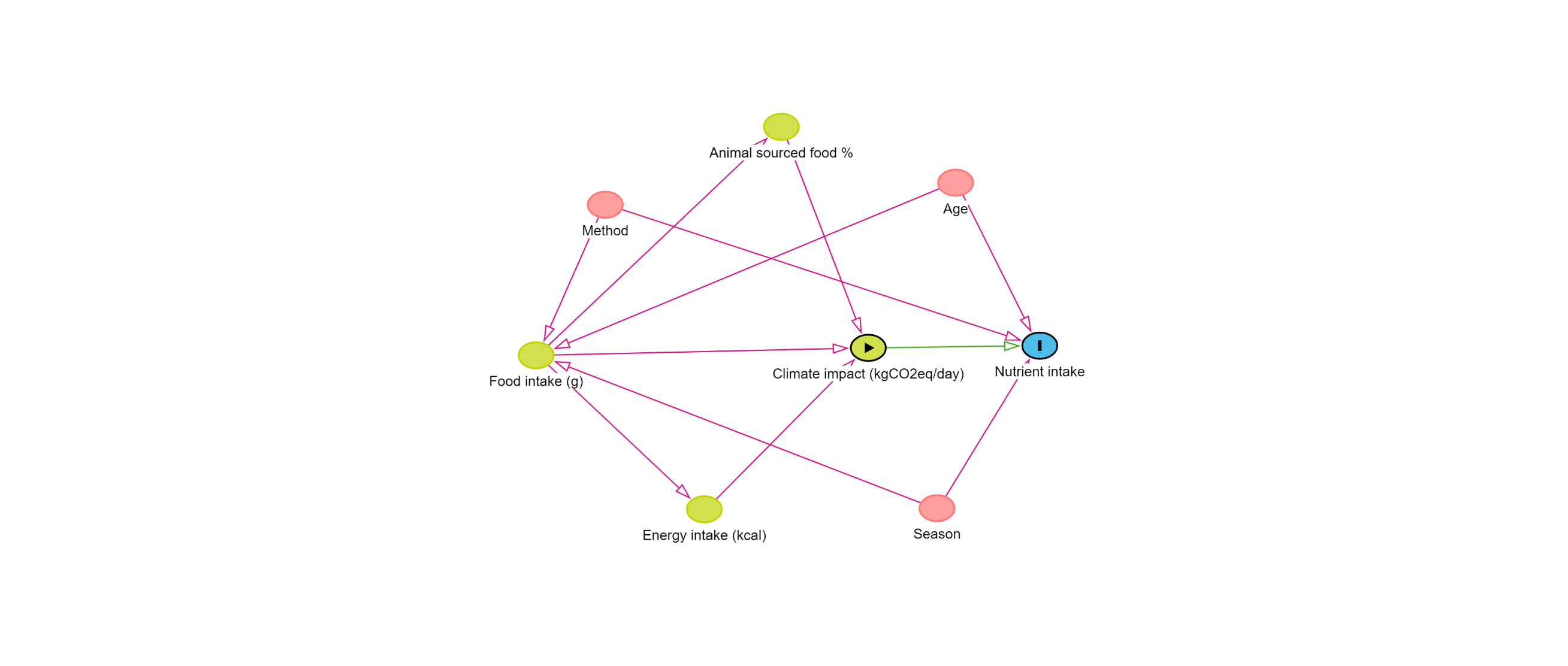


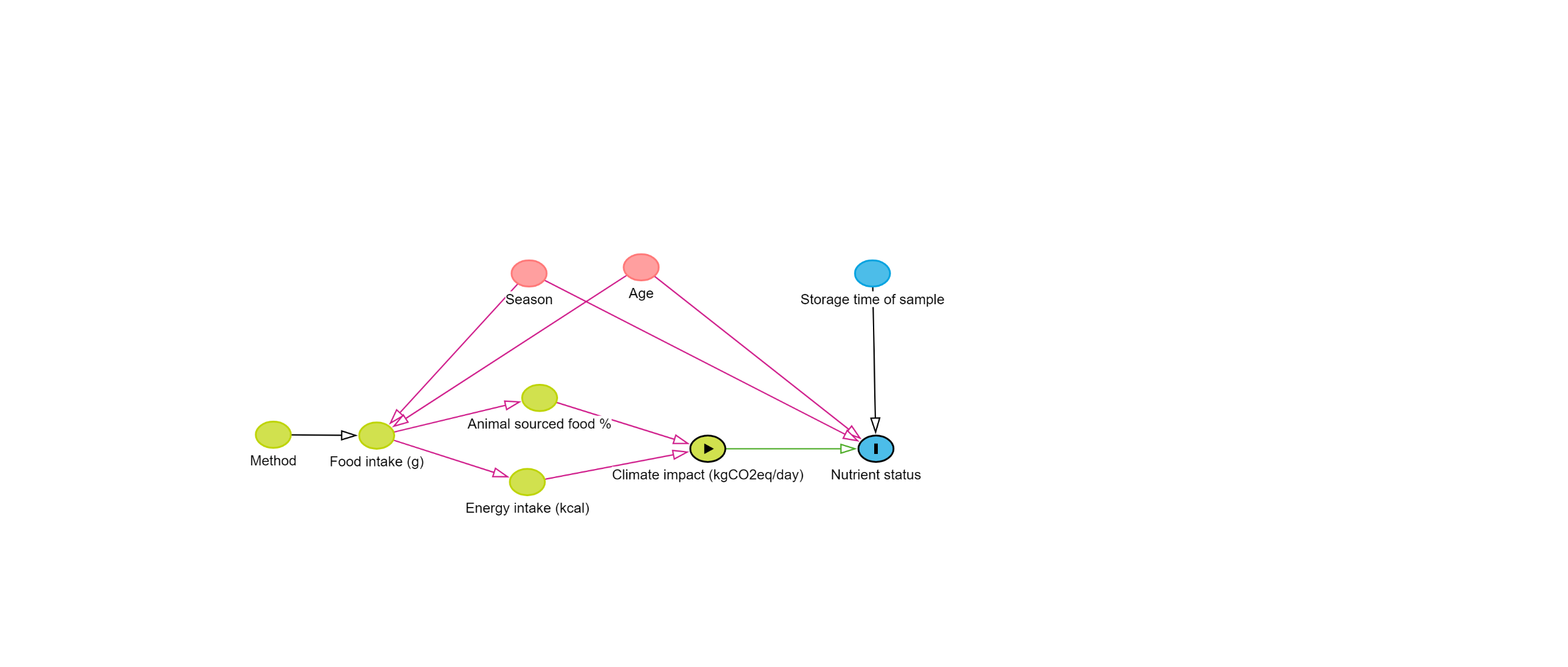


**Supplemental table 1**. Content of different food groups used in the calculations of dietary GHGE in the Malmö Diet and Cancer Study.

| **Superior food groups** | **Food groups** | **Included foods** |
| --- | --- | --- |
| Plant-based foods | Vegetables | Vegetables, root vegetables, potatoes, legumes, soy, vegetable juice |
|  | Fruit and berries | Fruit and berries (excluding juices), nuts^1^ |
|  | Cereals | Cereals, bread, rice, pasta |
|  | Added fats | Oils, margarines, mayonnaise, dressings |
|  | Other foods | Ketchup, spices, stock, other foods |
| Animal-sourced foods | Red meat | Beef, pork, lamb, game, processed red meat |
|  | Poultry and egg | Poultry and egg |
|  | Seafood | Fish and shellfish |
|  | Dairy | Milk, fermented milk, cream, cheese, butter, milk-based margarines |
| Discretionary foods | Non-alcoholic drinks | Coffee, tea, soda, fruit juices, water |
|  | Alcoholic drinks | Beer, wine, spiritus |
|  | Sweet and snacks | Sugar, candy, crisps, jam, cakes, ice cream, sorbet |
| ^1.^ Nuts was included in the fruit and berries group since the mean consumption was very low in this cohort (1.81 grams per day), | | |

**Supplemental table 2**. Daily consumption of different food groups in grams and their contribution to proportion (%) of GHGE among 25,970 participants from the Malmö Diet and Cancer Study.

|  | **Grams per day by quintiles of kg CO_2_eq/day^1^** | | | | | | | | | |
| --- | --- | --- | --- | --- | --- | --- | --- | --- | --- | --- |
|  | **1** | | **2** | **3** | **4** | | **5** | | **All** | |
| **Females (n)** | 3,163 | | 3,163 | 3,164 | 3,163 | | 3,163 | | 15,816 | |
| Vegetables | 226 (7.3%) | | 256 (6.5%) | 275 (6.1%) | 301 (5.9%) | | 340 (5.4%) | | 279 (6.2%) | |
| Fruit, berries, nuts | 175 (7.4%) | | 198 (6.4%) | 203 (5.6%) | 218 (5.2%) | | 227 (4.3%) | | 204 (5.8%) | |
| Cereals | 107 (5.3%) | | 119 (4.5%) | 126 (4.1%) | 135 (3.8%) | | 146 (3.3%) | | 127 (4.2%) | |
| Red meat | 66 (27%) | | 83 (30%) | 96 (32%) | 106 (34%) | | 135 (39%) | | 97 (32%) | |
| Poultry and egg | 30 (2.9%) | | 34 (2.5%) | 37 (2.3%) | 40 (2.1%) | | 44 (1.8%) | | 37 (2.3%) | |
| Seafood | 34 (6.1%) | | 39 (6.8%) | 42 (7.4%) | 45 (7.9%) | | 51 (9.0%) | | 42 (7.4%) | |
| Dairy | 312 (25%) | | 383 (25%) | 420 (24%) | 449 (23%) | | 510 (22%) | | 415 (24%) | |
| Added fats | 20 (1.6%) | | 22 (1.3%) | 24 (1.2%) | 25 (1.1%) | | 26 (1.0%) | | 24 (1.3%) | |
| Non-alcoholic drinks | 1445 (8%) | | 1509 (8%) | 1575 (8%) | 1660 (7%) | | 1756 (7%) | | 1589 (8%) | |
| Alcoholic drinks | 79 (3.5%) | | 111 (4%) | 130 (4.1%) | 153 (4.2%) | | 191 (4.2%) | | 133 (4%) | |
| Sweet and snacks | 71 (5.3%) | | 81 (4.9%) | 85 (4.6%) | 93 (4.4%) | | 102 (4%) | | 86 (4.6%) | |
| Other foods | 16 (0.6%) | | 21 (0.6%) | 21 (0.5%) | 22 (0.5%) | | 25 (0.4%) | | 21 (0.5%) | |
|  |  | |  |  |  | |  | |  | |
| **Males (n)** | 2,030 | | 2,031 | 2,031 | 2,031 | | 2,031 | | 10,154 | |
| Vegetables | 266 (6.3%) | | 299 (5.6%) | 319 (5.2%) | 342 (4.8%) | | 395 (4.4%) | | 324 (5.3%) | |
| Fruit, berries, nuts | 150 (5.1%) | | 168 (4.4%) | 171 (3.8%) | 176 (3.3%) | | 185 (2.7%) | | 170 (3.9%) | |
| Cereals | 154 (6.1%) | | 173 (5.3%) | 186 (4.9%) | 197 (4.4%) | | 218 (3.8%) | | 186 (4.9%) | |
| Red meat | 99 (31%) | | 123 (35%) | 141 (37%) | 163 (40%) | | 215 (45%) | | 148 (38%) | |
| Poultry and egg | 37 (2.8%) | | 40 (2.3%) | 42 (2.1%) | 47 (2.0%) | | 52 (1.7%) | | 43 (2.2%) | |
| Seafood | 43 (5.7%) | | 47 (6.3%) | 50 (6.3%) | 51 (6.4%) | | 59 (7.1%) | | 50 (6.4%) | |
| Dairy | 348 (23%) | | 420 (22%) | 466 (22%) | 514 (21%) | | 577 (19%) | | 465 (22%) | |
| Added fats | 29 (1.7%) | | 33 (1.5%) | 35 (1.4%) | 36 (1.3%) | | 37 (1%) | | 34 (1.4%) | |
| Non-alcoholic drinks | 1282 (7%) | | 1323 (6%) | 1356 (6%) | 1442 (6%) | | 1551 (5%) | | 1391 (6%) | |
| Alcoholic drinks | 179 (5.8%) | | 252 (6.4%) | 292 (6.4%) | 325 (6.2%) | | 406 (6%) | | 291 (6.2%) | |
| Sweet and snacks | 87 (5%) | | 97 (4.5%) | 103 (4.3%) | 106 (3.8%) | | 116 (3.3%) | | 102 (4.2%) | |
| Other foods | 18 (0.5%) | | 21 (0.4%) | 22 (0.4%) | 23 (0.3%) | | 26 (0.3%) | | 22 (0.4%) | |
|  |  |  | |  | |  | |  | |  |
| ^1.^ Quintiles of dietary GHGE per day for females/males 1: 1: <4.1/<5.0, 2: 4.1-4.8/5.0-5.9, 3: 4.8-5.6/5.9-6.9, 4: 5.7-6.5/6.9-8.2, 5: >6.5/>8.2 kg CO_2_eq. | | | | | | | | | | |

**Supplemental table 3**. Nutritional supplement intake for 25,970 participants from the Malmö Diet and Cancer Study1. Values are adjusted for dietary assessment version, season, and age.

|  | **Quintiles of dietary climate impact (kg CO_2_eq/day)**^2^ | | | | |  |  |
| --- | --- | --- | --- | --- | --- | --- | --- |
|  | **1** | **2** | **3** | **4** | **5** | **β** | **p**^3^ |
|  |  |  |  |  |  |  |  |
| **Female (n)** | 3163 | 3163 | 3164 | 3163 | 3163 |  |  |
| Vitamin A (RE^4^) | 212 (735) | 224 (730) | 232 (730) | 245 (728) | 280 (734) | 15.8 | <0.001 |
| Vitamin D (μg) | 1.2 (3.2) | 1.4 (3.2) | 1.4 (3.2) | 1.5 (3.2) | 1.6 (3.2) | 0.09 | <0.001 |
| Vitamin E (a-TE^5^) | 5.7 (30.5) | 5.7 (30.3) | 6.5 (30.3) | 7.1 (30.2) | 6.5 (30.5) | 0.3 | 0.084 |
| Thiamine (mg) | 1.1 (7.6) | 0.8 (7.5) | 1 (7.5) | 1.1 (7.5) | 1.3 (7.6) | 0.05 | 0.223 |
| Riboflavin (mg) | 0.8 (4.9) | 0.8 (4.9) | 1 (4.9) | 1 (4.9) | 1.2 (4.9) | 0.1 | <0.001 |
| Niacin (NE^6^) | 6.2 (38.4) | 6.4 (38.2) | 7.2 (38.2) | 7.4 (38.1) | 8.8 (38.4) | 0.62 | 0.005 |
| Vitamin B6 (mg) | 1.5 (17.2) | 1.9 (17.1) | 2.8 (17.1) | 2.1 (17.1) | 2 (17.2) | 0.12 | 0.205 |
| Folate (μg) | 56 (308) | 50 (306) | 56 (306) | 48 (305) | 48 (307) | -1.76 | 0.312 |
| Vitamin B12 (μg) | 3.4 (148) | 10.7 (147) | 9.6 (147) | 3.2 (147) | 7.6 (148) | 0.1 | 0.904 |
| Vitamin C (mg) | 67 (281) | 62 (279) | 76 (279) | 80 (278) | 83 (281) | 4.89 | 0.002 |
| Calcium (mg) | 23 (112) | 23 (112) | 21 (112) | 21 (111) | 24 (112) | 0.07 | 0.917 |
| Phosphorus (mg) | 3.3 (23.5) | 2.6 (23.4) | 2.8 (23.3) | 3.3 (23.3) | 3.3 (23.5) | 0.07 | 0.596 |
| Magnesium (mg) | 8.9 (47) | 9 (47) | 7.8 (47) | 9.5 (46) | 9.5 (47) | 0.16 | 0.546 |
| Potassium (g) | 0.01 (0.06) | 0.01 (0.07) | 0.01 (0.07) | 0.01 (0.05) | 0.01 (0.07) | 0.00 | 0.118 |
| Zinc (mg) | 1.8 (5.7) | 2 (5.6) | 2.1 (5.6) | 2.2 (5.6) | 2.1 (5.7) | 0.09 | 0.032 |
| Selenium (μg) | 7 (22.9) | 8 (22.8) | 8.9 (22.7) | 9.4 (22.7) | 9.2 (22.9) | 0.60 | <0.001 |
| Iron (mg) | 2.8 (12.3) | 2.9 (12.2) | 3.2 (12.2) | 3.2 (12.2) | 3.2 (12.3) | 0.12 | 0.089 |
|  |  |  |  |  |  |  |  |
| **Males (n)** | 2030 | 2031 | 2031 | 2031 | 2031 |  |  |
| Vitamin A (RE^4^) | 138 (543) | 152 (534) | 158 (532) | 147 (535) | 163 (543) | 4.5 | 0.241 |
| Vitamin D (μg) | 0.9 (2.6) | 0.9 (2.6) | 0.9 (2.6) | 0.8 (2.6) | 0.9 (2.6) | 0.01 | 0.517 |
| Vitamin E (a-TE^5^) | 3.7 (23) | 3.8 (22.6) | 3.2 (22.5) | 4.1 (22.7) | 3.7 (23) | 0.03 | 0.138 |
| Thiamine (mg) | 0.4 (7.5) | 0.9 (7.4) | 0.7 (7.3) | 0.8 (7.4) | 1.2 (7.5) | 0.16 | 0.004 |
| Riboflavin (mg) | 0.4 (3.9) | 0.5 (3.8) | 0.6 (3.8) | 0.7 (3.8) | 0.8 (3.9) | 0.09 | 0.218 |
| Niacin (NE^6^) | 6.8 (101.9) | 9.3 (100.3) | 5.9 (99.8) | 4.8 (100.5) | 6.6 (101.9) | -0.48 | 0.505 |
| Vitamin B6 (mg) | 0.7 (6.2) | 0.7 (6.1) | 0.7 (6.1) | 0.8 (6.2) | 1 (6.2) | 0.07 | 0.111 |
| Folate (μg) | 46 (325) | 40 (320) | 31 (318) | 31 (320) | 43 (325) | -1.5 | 0.515 |
| Vitamin B12 (μg) | 3.5 (52) | 2.8 (51) | 3.6 (51) | 2.8 (52) | 3.4 (52) | -0.01 | 0.988 |
| Vitamin C (mg) | 48 (235) | 54 (231) | 40 (230) | 42 (232) | 51 (235) | -0.71 | 0.063 |
| Calcium (mg) | 7 (56) | 6 (56) | 8 (55) | 7 (56) | 8 (56) | 0.29 | 0.464 |
| Phosphorus (mg) | 1.8 (31.9) | 2.9 (31.4) | 1.7 (31.2) | 1.6 (31.4) | 2.4 (31.9) | -0.01 | 0.962 |
| Magnesium (mg) | 3.7 (37) | 4.4 (36) | 5.3 (36) | 5 (36) | 6.2 (37) | 0.56 | 0.033 |
| Potassium (g) | 0.01 (0.05) | 0.01 (0.06) | 0.01 (0.07) | 0.01 (0.06) | 0.01 (0.06) | 0.00 | 0.158 |
| Zinc (mg) | 1.2 (4.1) | 1.3 (4) | 1.2 (4) | 1.2 (4) | 1.2 (4.1) | -0.02 | 0.607 |
| Selenium (μg) | 5.5 (18.5) | 5.6 (18.2) | 5.4 (18.1) | 5 (18.3) | 4.8 (18.5) | -0.19 | 0.143 |
| Iron (mg) | 2.3 (9.2) | 1.7 (9.0) | 1.6 (9.0) | 1.4 (9.0) | 1.4 (9.1) | -0.20 | 0.002 |
|  |  |  |  |  |  |  |  |
| ^1.^ Values are adjusted estimated means (SD) (based on general linear model) or percentages. ^2.^ Quintiles of dietary greenhouse gas emissions per day for females 1:<4.1. 2: 4.1-4.8. 3: 4.8-5.56. 4: 5.5-6.5. 5: >6.5 kg CO_2_eq. ^3.^ P-trend for general linear model. ^4.^ Retinol-equivalents. ^5.^ Alpha-tocopherol equivalents ^6.^ Niacin equivalents. | | | | | | | |

**Supplemental table 4**. Participant characteristics according to quintiles of dietary climate impact (kg CO_2_eq/day) in subgroups of participants from the Malmö Diet and Cancer Study.

|  | **Quintiles of dietary climate impact (kg CO_2_eq/day)^1,2^** | | | | |  |
| --- | --- | --- | --- | --- | --- | --- |
|  | **1** | **2** | **3** | **4** | **5** | |
| **1. Females (n)**^3^ | 214 | 213 | 217 | 221 | 250 | |
| Age (years) | 58.1 (7.4) | 57.8 (8.1) | 56.8 (7.1) | 56 (7.1) | 54.5 (6.4) | |
| BMI^2^ | 26 (4.5) | 25.9 (4.7) | 25.4 (3.7) | 24.9 (4.1) | 25.2 (3.8) | |
| BMI above 25 % | 52.3 | 49.8 | 49.3 | 39.8 | 45.6 | |
| Current smokers % | 30.8 | 27.7 | 26.7 | 24.0 | 34.0 | |
| High alcohol consumption^4^ % | 0.0 | 0.0 | 3.2 | 2.7 | 9.6 | |
| High physical activity^5^ % | 20.1 | 13.1 | 24.1 | 23.1 | 16.0 | |
| University degree % | 13.1 | 11.3 | 19.8 | 24.1 | 24.8 | |
| Prevalent diabetes % | 5.1 | 1.9 | 0.9 | 4.1 | 1.6 | |
| Prevalent cancer % | 12.1 | 13.6 | 12.9 | 13.6 | 15.2 | |
| Prevalent CVD % | 0.0 | 2.3 | 0.5 | 0.5 | 0.0 | |
| Energy intake, kcal/day | 1606 (317) | 1830 (331) | 2034 (372) | 2177 (398) | 2494 (529) | |
| Fat E% | 37 (5.6) | 36.7 (5.5) | 37.8 (6.2) | 38.2 (6) | 39.1 (5.7) | |
| Saturated fat E% | 15.5 (3.5) | 15.8 (3.5) | 16.3 (3.8) | 16.7 (3.9) | 17.3 (3.5) | |
| Unsaturated fat E% | 19.2 (3.1) | 18.6 (3) | 19.1 (3.2) | 19 (3.2) | 19.4 (3.2) | |
| Protein E% | 14.9 (2.5) | 15.6 (2.2) | 15.5 (2.4) | 15.8 (2.5) | 16.2 (2.5) | |
| Carbohydrate E% | 48.1 (5.7) | 47.7 (5.4) | 46.7 (6) | 46 (5.7) | 44.8 (5.9) | |
| Dietary fibre, g | 16.2 (6.5) | 17.9 (5.3) | 19.2 (5.4) | 20.8 (6.3) | 21.8 (7.4) | |
| Dietary fibre g/1,000 kcal | 10.3 (3.4) | 10.2 (2.9) | 9.8 (2.6) | 10 (2.9) | 9.2 (2.5) | |
|  |  |  |  |  |  | |
| **2. Females (n)**^6^ | 295 | 270 | 296 | 296 | 321 | |
| Age (years) | 58.7 (7.3) | 57.6 (7.7) | 57.8 (7) | 57 (7) | 55 (6.5) | |
| BMI | 26.4 (4.5) | 25.7 (4.6) | 25.8 (4.2) | 25.3 (4) | 25.4 (4.4) | |
| BMI above 25 % | 54.6 | 54.1 | 50.3 | 44.9 | 46.1 | |
| Current smokers % | 23.1 | 27.0 | 27.7 | 26.0 | 32.7 | |
| High alcohol consumption^4^ % | 0.3 | 0.7 | 1.7 | 2.7 | 6.2 | |
| High physical activity^5^ % | 16.1 | 15.2 | 23.4 | 20.1 | 19.7 | |
| University degree % | 7.2 | 16.3 | 13.9 | 21.8 | 20.4 | |
| Prevalent diabetes % | 3.4 | 3.7 | 2.0 | 3.4 | 4.7 | |
| Prevalent cancer % | 7.5 | 8.5 | 6.1 | 6.1 | 8.1 | |
| Prevalent CVD % | 0.7 | 1.9 | 1.0 | 1.7 | 0.3 | |
| Energy intake, kcal/day | 1618 (340) | 1837 (296) | 2022 (357) | 2209 (371) | 2535 (524) | |
| Fat E% | 36.5 (5.9) | 37.4 (5.6) | 37.4 (6.2) | 38.5 (5.6) | 39.1 (5.5) | |
| Saturated fat E% | 15.2 (3.6) | 16.2 (3.5) | 16.1 (3.6) | 17 (3.8) | 17.3 (3.8) | |
| Unsaturated fat E% | 18.9 (3.2) | 18.9 (3.1) | 18.9 (3.2) | 19.1 (2.9) | 19.4 (2.9) | |
| Protein E% | 15 (2.5) | 15.5 (2.2) | 15.6 (2.5) | 15.7 (2.5) | 16.1 (2.4) | |
| Carbohydrate E% | 48.5 (5.8) | 47.1 (5.6) | 47.1 (6) | 45.8 (5.2) | 44.8 (5.5) | |
| Dietary fibre, g | 16.3 (5.9) | 17.7 (5.5) | 19.2 (5.6) | 20.3 (6.1) | 21.9 (6.8) | |
| Dietary fibre g/1,000 kcal | 10.4 (3.3) | 10 (2.8) | 9.8 (2.5) | 9.5 (2.6) | 9 (2.4) | |
|  |  |  |  |  |  | |
| **3. Males (n)** | 350 | 334 | 310 | 301 | 254 | |
| Age (years) | 64.1 (6.2) | 62.9 (6) | 62.1 (6.2) | 60.4 (6) | 58 (6) | |
| BMI | 26.5 (3.5) | 26.1 (3.3) | 25.8 (3.1) | 26.5 (3.5) | 26.1 (3.4) | |
| BMI above 25 % | 63.3 | 63.7 | 62.9 | 66.1 | 61.0 | |
| Current smokers % | 21.7 | 24.0 | 21.0 | 27.9 | 33.9 | |
| High alcohol consumption^4^ % | 2.3 | 2.4 | 6.8 | 10.0 | 13.8 | |
| High physical activity^5^ % | 20.6 | 22.8 | 22.1 | 22.6 | 19.7 | |
| University degree % | 10.9 | 10.8 | 13.9 | 16.9 | 16.1 | |
| Prevalent diabetes % | 5.4 | 6.9 | 5.2 | 7.0 | 5.1 | |
| Prevalent cancer % | 5.1 | 4.5 | 4.5 | 3.3 | 1.6 | |
| Prevalent CVD % | 7.1 | 6.0 | 2.6 | 3.3 | 4.3 | |
| Energy intake, kcal/day | 2043 (407) | 2389 (406) | 2641 (417) | 2853 (523) | 3346 (740) | |
| Fat E% | 37.4 (6.2) | 38.5 (6.2) | 39.5 (6.2) | 39.8 (6.2) | 40.9 (6.4) | |
| Saturated fat E% | 15.6 (3.6) | 16.3 (3.9) | 16.9 (4) | 17.2 (3.9) | 17.9 (4.4) | |
| Unsaturated fat E% | 19.5 (3.6) | 19.8 (3.4) | 20.2 (3.4) | 20.2 (3.4) | 20.5 (3.2) | |
| Protein E% | 14.8 (2.4) | 14.9 (2.2) | 14.9 (2.1) | 15.5 (2.4) | 15.8 (2.6) | |
| Carbohydrate E% | 47.8 (6.5) | 46.6 (6) | 45.6 (5.8) | 44.7 (6) | 43.2 (6) | |
| Dietary fibre, g | 18.4 (7.1) | 20.7 (7.1) | 22.1 (6.6) | 22.7 (7.7) | 24.9 (8.5) | |
| Dietary fibre g/1,000 kcal | 9.3 (3.0) | 9.0 (2.6) | 8.8 (2.3) | 8.4 (2.5) | 7.9(2.3) | |
| First group consists of females with analyses of Vitamin D, Zinc, and Selenium, and second group of on 1478 females with valid analyses on Folate and third group of 1549 males with analyses on Vitamin D. ^1.^ Quintiles of dietary greenhouse gas emissions per day for females/males 1: 1: <4.1/<5.0, 2: 4.1-4.8/5.0-5.9, 3: 4.8-5.6/5.9-6.9, 4: 5.7-6.5/6.9-8.2, 5: >6.5/>8.2 kg CO_2_eq. ^2.^ Values are means (SD) or percentages. ^3.^ Based on 1115 females with valid analyses on all the following serum variables: Vitamin D, Zinc and Selenium ^4.^ High alcohol consumption defined as above 30 g/d for females and above 40 g/d for males. ^5.^ Highest leisure time physical activity quintile. ^6.^ Based on 1478 females with valid analyses on folate. | | | | | | |

**Supplemental table 5**. Micronutrient intake for different subgroups with available data on micronutrient status from the Malmö Diet and Cancer Study^1^. Intake data was adjusted for dietary assessment version, season, and age. Recommendations for average requirement (AR) and recommended intake (RI) are from Nordic nutrition recommendations.

|  | **Quintiles of CO_2_eq/kg/day**^2^ | | | | |  |  |
| --- | --- | --- | --- | --- | --- | --- | --- |
|  | **1** | **2** | **3** | **4** | **5** | **β** | **P**^3^ |
| **Females** |  |  |  |  |  |  |  |
| **Vitamin D (n)** | *257* | *256* | *267* | *253* | *300* |  |  |
| D vitamin, (μg), total | 6.1 (4) | 7.3 (4) | 7.9 (4) | 8.7 (4) | 9.6 (4) | 0.833 | <0.001 |
| Dietary intake | 5.2 (2.4) | 5.9 (2.4) | 6.6 (2.4) | 6.9 (2.4) | 7.9 (2.4) | 0.649 | <0.001 |
| Dietary intake/1000 kcal (RI 5) | 3.3 (1.1) | 3.2 (1.1) | 3.3 (1.1) | 3.2 (1.1) | 3.2 (1.1) | -0.019 | 0.359 |
| Dietary intake above AR (7.5) | 13.0% | 19.6% | 33.1% | 37.9% | 51.7% | 0.096 | <0.001 |
| Dietary intake above RI (10) | 0.7% | 5.0% | 10.4% | 8.4% | 18.5% | 0.034 | <0.001 |
| Supplement^4^ | 1 (3.2) | 1.4 (3.2) | 1.2 (3.2) | 1.8 (3.2) | 1.7 (3.2) | 0.185 | 0.003 |
| **Selenium (n)** | *356* | *377* | *371* | *407* | *435* |  |  |
| Selenium (μg), total | 34.1 (25.4) | 38.3 (25.4) | 43 (25.3) | 48.4 (25.3) | 53.2 (25.5) | 4.83 | <0.001 |
| Dietary intake | 26.6 (10.1) | 31.3 (10.1) | 33.9 (10.1) | 36.7 (10.1) | 43.1 (10.2) | 3.86 | <0.001 |
| Dietary intake/1000 kcal (RI 26) | 17 (5.4) | 17.3 (5.3) | 17.2 (5.3) | 17.1 (5.3) | 17.8 (5.4) | 0.125 | 0.150 |
| Dietary intake above AR (30) | 27.8% | 50.6% | 65.3% | 74.1% | 87.5% | 0.142 | <0.001 |
| Dietary intake above RI (50) | 0% | 0.3% | 0% | 0% | 2.3% | 0.055 | <0.001 |
| Supplement^7^ | 7.6 (22.8) | 7 (22.8) | 9.1 (22.7) | 11.7 (22.7) | 10.1 (22.9) | 0.968 | 0.009 |
| **Zinc (n)** | *356* | *377* | *371* | *407* | *435* |  |  |
| Zinc (mg), total | 9.1 (5.4) | 11 (5.4) | 11.7 (5.4) | 13.3 (5.4) | 15.1 (5.4) | 1.417 | <0.001 |
| Dietary intake | 7.4 (1.9) | 8.9 (1.9) | 9.8 (1.9) | 11 (1.9) | 12.8 (1.9) | 1.299 | <0.001 |
| Dietary intake/1000 kcal (RI 3.8) | 4.7 (0.8) | 4.9 (0.8) | 4.9 (0.8) | 5.1 (0.8) | 5.2 (0.8) | 0.128 | <0.001 |
| Dietary intake above AR (5) | 95% | 100% | 100% | 100% | 100% | 0.009 | <0.001 |
| Dietary intake above RI (7) | 58.2% | 91.1% | 96.8% | 99.1% | 99.9% | 0.088 | <0.001 |
| Supplement^8^ | 1.8 (4.9) | 2.1 (4.9) | 1.9 (4.9) | 2.3 (4.9) | 2.2 (4.9) | 0.119 | 0.317 |
| **Folate (n)** | *295* | *270* | *296* | *296* | *321* |  |  |
| Folate (μg), total | 240 (180) | 248 (179) | 275 (179) | 293 (179) | 311 (181) | 18.84 | <0.001 |
| Dietary intake | 190 (65) | 210 (65) | 233 (65) | 249 (65) | 279 (65) | 21.65 | <0.001 |
| Dietary intake/1000 kcal (RI 188) | 119 (30) | 115 (30) | 117 (30) | 114 (30) | 111 (30) | -1.708 | 0.002 |
| Dietary intake above AR (1) | 37.4% | 51.3% | 70.7% | 79.0% | 86.0% | 0.124 | <0.001 |
| Dietary intake above RI (1.2)^9^ | 4.6% | 7.3% | 12.4% | 18.7% | 32.1% | 0.067 | <0.001 |
| Supplement^10^ | 49.4 (165.4) | 38 (164.7) | 41.5 (164.9) | 44.2 (164.9) | 32.6 (166.3) | -2.81 | 0.353 |
|  |  |  |  |  |  |  |  |
| **Males** |  |  |  |  |  |  |  |
| **Vitamin D (n)** | 354 | 340 | 315 | 304 | 257 |  |  |
| D vitamin, total intake | 8.2 (4.5) | 9.4 (4.5) | 9.8 (4.4) | 10.6 (4.5) | 11.9 (4.6) | 0.862 | <0.001 |
| Dietary intake | 7 (3.6) | 8.3 (3.5) | 8.9 (3.5) | 9.6 (3.5) | 10.9 (3.6) | 0.906 | <0.001 |
| Dietary intake per 1000 kcal | 3.5 (1.2) | 3.5 (1.2) | 3.3 (1.2) | 3.3 (1.2) | 3.3 (1.2) | -0.056 | 0.011 |
| Dietary intake above AR (%) | 13% | 19.6% | 33.1% | 37.9% | 51.7% | 0.095 | <0.001 |
| Dietary intake above RI (%) | 13.4% | 23.2% | 31.0% | 37.5% | 54.1% | 0.094 | <0.001 |
| Supplement | 1.2 (2.8) | 1.1 (2.7) | 1 (2.7) | 1 (2.7) | 1 (2.8) | -0.044 | 0.4 |
| ^1.^ Values are adjusted estimated means (SD) (based on general linear model) or percentages. ^2.^ Quintiles of dietary greenhouse gas emissions per day for females 1:<4.08, 2: 4.08-4.82, 3: 4.82-5.55, 4: 5.55-6.50, 5: >6.50 kg CO_2_eq. ^3.^ P-trend for general linear model. | | | | | | | |

**Supplemental table 6a**. Micronutrient intakes across quintiles of dietary climate impact (GHGE/1000 kcal) for 15,816 females from the Malmö Diet and Cancer Study1. AR and RI are from NNR [1].

|  | **Quintiles of dietary climate impact (kg CO_2_eq/1000 kcal)** ^2^ | | | | |  |  |
| --- | --- | --- | --- | --- | --- | --- | --- |
|  | **1** | **2** | **3** | **4** | **5** | **β** | **p**^3^ |
|  |  |  |  |  |  |  |  |
| **Vitamin A (RE**^4^**)**  *Dietary intake* | 847 (1186) | 850 (1179) | 846 (1177) | 856 (1179) | 885 (1188) | 8.09 | 0.007 |
| **Vitamin D (μg)**  *Dietary intake* | 3.2 (2.5) | 3.2 (2.5) | 3.2 (2.5) | 3.3 (2.5) | 3.3 (2.5) | 0.02 | 0.002 |
| **Vitamin E (a-TE**^5^**)**  *Dietary intake* | 4.3 (2.8) | 4.2 (2.8) | 4.2 (2.7) | 4.3 (2.8) | 4.4 (2.8) | 0.03 | <0.001 |
| **Thiamine (mg)**  *Dietary intake* | 0.6 (0.2) | 0.6 (0.2) | 0.6 (0.2) | 0.6 (0.2) | 0.6 (0.2) | 0.01 | <0.001 |
| **Riboflavin (mg)**  *Dietary intake* | 0.8 (0.5) | 0.8 (0.5) | 0.9 (0.5) | 0.9 (0.5) | 0.9 (0.5) | 0.03 | <0.001 |
| **Niacin (NE**^6^**)**  *Dietary intake* | 14.2 (5.1) | 15.1 (5) | 15.8 (5) | 16.5 (5) | 18.2 (5.1) | 0.93 | <0.001 |
| **Vitamin B6 (mg)**  *Dietary intake* | 0.8 (0.4) | 0.9 (0.4) | 0.9 (0.4) | 0.9 (0.4) | 1 (0.5) | 0.04 | <0.001 |
| **Folate (μg),**  *Dietary intake* | 108 (69) | 111 (68) | 115 (68) | 118 (68) | 124 (69) | 4.06 | <0.001 |
| **Vitamin B12 (μg)**  *Dietary intake* | 2.6 (5.5) | 2.9 (5.4) | 3 (5.4) | 3 (5.4) | 3.3 (5.5) | 0.16 | <0.001 |
| **Vitamin C (mg)**  *Dietary intake* | 47 (64) | 51 (63) | 56 (63) | 59 (63) | 63 (64) | 3.89 | <0.001 |
| **Calcium (mg)**  *Dietary intake* | 504 (327) | 531 (325) | 548 (325) | 554 (325) | 557 (327) | 12.92 | <0.001 |
| **Phosphorus (mg)**  *Dietary intake* | 645 (277) | 682 (275) | 709 (275) | 724 (275) | 757 (277) | 26.44 | <0.001 |
| **Magnesium (mg)**  *Dietary intake* | 149 (56.9) | 153.1 (56.6) | 158.6 (56.5) | 161.8 (56.6) | 170 (57) | 5.06 | <0.001 |
| **Potassium (g)**  *Dietary intake* | 1.4 (0.7) | 1.5 (0.7) | 1.6 (0.7) | 1.6 (0.7) | 1.8 (0.7) | 0.08 | <0.001 |
| **Iron (mg)**  *Dietary intake* | 6.7 (2.8) | 6.6 (2.8) | 6.6 (2.8) | 6.7 (2.8) | 7 (2.8) | 0.08 | <0.001 |
| **Zinc (mg)**  *Dietary intake* | 4.4 (1.6) | 4.7 (1.6) | 4.9 (1.5) | 5.2 (1.6) | 5.7 (1.6) | 0.31 | <0.001 |
| **Selenium (μg)**  *Dietary intake* | 14.8 (11.4) | 16.2 (11.3) | 17.4 (11.3) | 18.1 (11.3) | 19.9 (11.4) | 1.20 | <0.001 |
| Values are means (SD) and are adjusted for dietary assessment version, season, and age ^2.^ Quintiles of dietary greenhouse gas emissions per 1000 kcal for females 1: <2.2. 2: 2.2-2.5. 3: 2.5-2.7. 4: 2.7-3.1. 5: >3.1 kg CO_2_eq. ^3.^ P-trend for general linear model. ^4.^ Retinol-equivalents. ^5.^ Alpha-tocopherol equivalents ^6.^ Niacin equivalents. | | | | | | | |

**Supplemental table 6b**. Micronutrient intakes across quintiles of dietary climate impact (GHGE/1000 kcal) for 10,154 males from the Malmö Diet and Cancer Study1. AR and RI are from NNR [1].

|  | **Quintiles of dietary climate impact (kg CO_2_eq/1000 kcal)** ^2^ | | | | |  |  |
| --- | --- | --- | --- | --- | --- | --- | --- |
|  | **1** | **2** | **3** | **4** | **5** | **β** | **p**^3^ |
|  |  |  |  |  |  |  |  |
| **Vitamin A (RE**^4^**)**  *Dietary intake* | 827 (1235) | 805 (1227) | 789 (1223) | 802 (1225) | 774 (1236) | -10.79 | 0.006 |
| **Vitamin D (μg)**  *Dietary intake* | 3.4 (2.7) | 3.4 (2.7) | 3.4 (2.7) | 3.3 (2.7) | 3.3 (2.7) | -0.04 | <0.001 |
| **Vitamin E (a-TE**^5^**)**  *Dietary intake* | 4.0 (2.9) | 3.9 (2.9) | 3.9 (2.9) | 3.9 (2.9) | 4.0 (2.9) | 0.00 | 0.787 |
| **Thiamine (mg)**  *Dietary intake* | 0.6 (0.3) | 0.6 (0.2) | 0.6 (0.2) | 0.6 (0.2) | 0.6 (0.3) | 0.00 | 0.007 |
| **Riboflavin (mg)**  *Dietary intake* | 0.8 (0.5) | 0.8 (0.5) | 0.8 (0.5) | 0.8 (0.5) | 0.8 (0.5) | 0.02 | <0.001 |
| **Niacin (NE**^6^**)**  *Dietary intake* | 14.2 (5) | 14.8 (4.9) | 15.4 (4.9) | 16.1 (4.9) | 17.6 (5) | 0.81 | <0.001 |
| **Vitamin B6 (mg)**  *Dietary intake* | 0.8 (0.4) | 0.8 (0.4) | 0.8 (0.4) | 0.9 (0.4) | 0.9 (0.4) | 0.03 | <0.001 |
| **Folate (μg),**  *Dietary intake* | 97 (60) | 97 (59) | 98 (59) | 100 (59) | 102 (60) | 1.37 | <0.001 |
| **Vitamin B12 (μg)**  *Dietary intake* | 2.8 (5.5) | 2.9 (5.5) | 3 (5.5) | 3.1 (5.5) | 3.2 (5.5) | 0.10 | <0.001 |
| **Vitamin C (mg)**  *Dietary intake* | 33 (51) | 36 (50) | 37 (50) | 39 (50) | 41 (51) | 1.98 | <0.001 |
| **Calcium (mg)**  *Dietary intake* | 424 (315) | 450 (313) | 461 (312) | 466 (312) | 459 (315) | 8.55 | <0.001 |
| **Phosphorus (mg)**  *Dietary intake* | 610 (270) | 641 (268) | 658 (267) | 676 (268) | 697 (270) | 21.01 | <0.001 |
| **Magnesium (mg)**  *Dietary intake* | 141 (51.7) | 143.4 (51.4) | 147 (51.2) | 150.2 (51.3) | 154.7 (51.8) | 3.43 | <0.001 |
| **Potassium (g)**  *Dietary intake* | 1.3 (0.6) | 1.3 (0.6) | 1.4 (0.6) | 1.5 (0.6) | 1.5 (0.6) | 0.06 | <0.001 |
| **Iron (mg)**  *Dietary intake* | 7.2 (3.2) | 6.8 (3.2) | 6.8 (3.2) | 6.9 (3.2) | 7.1 (3.2) | -0.01 | 0.438 |
| **Zinc (mg)**  *Dietary intake* | 4.3 (1.6) | 4.6 (1.6) | 4.8 (1.6) | 5.1 (1.6) | 5.6 (1.6) | 0.31 | <0.001 |
| **Selenium (μg)**  *Dietary intake* | 14.3 (11.2) | 15.3 (11.1) | 16.1 (11.1) | 16.7 (11.1) | 17.9 (11.2) | 0.86 | <0.001 |
| Values are means (SD) and are adjusted for dietary assessment version, season, and age ^2.^ Quintiles of dietary greenhouse gas emissions per 1000 kcal for males 1: <2.1. 2: 2.1-2.3. 3: 2.3-2.6. 4: 2.6-3.0 5: >8.7 kg CO_2_eq. ^3.^ P-trend for general linear model. ^4.^ Retinol-equivalents. ^5.^ Alpha-tocopherol equivalents ^6.^ Niacin equivalents. | | | | | | | |

**Supplemental table 7**. Micronutrient status for different subgroups from the Malmö Diet and Cancer Study by total intake GHGE/day and CO2eq/1000 kcal, as continuous variables and as quintiles. Model is adjusted for age, season and storage time of sample for all but Hb.

| **Coefficients for micronutrient status on different exposures of GHGE** | | | | | | | | |
| --- | --- | --- | --- | --- | --- | --- | --- | --- |
|  | GHGE/day | | Quintiles GHGE/day | | GHGE/1000 kcal | | Quintiles of GHGE/1000 kcal | |
|  | **β** | **p** | **β** | **p** | **β** | **p** | **β** | **p** |
| **Females** |  |  |  |  |  |  |  |  |
|  |  |  |  |  |  |  |  |  |
| Vitamin D (25OHD_3_ nmol/L) | -0.32 | 0.492 | -0.31 | 0.560 | -1.90 | 0.118 | -0.80 | 0.135 |
| Below reference (50 nmol/L) | 0.07 | 0.287 | 0.06 | 0.441 | 0.28 | 0.114 | 0.08 | 0.367 |
|  |  |  |  |  |  |  |  |  |
| Serum selenium (ng/ml) | 0.19 | 0.425 | 0.01 | 0.956 | 0.54 | 0.384 | 0.12 | 0.652 |
| Below reference (63 ng/ml) | 0.05 | 0.570 | 0.14 | 0.178 | -0.16 | 0.535 | -0.03 | 0.785 |
|  |  |  |  |  |  |  |  |  |
| Serum zinc (μg/L) | -0.01 | 0.785 | -0.05 | 0.078 | 0.01 | 0.940 | -0.03 | 0.259 |
| Below reference (10.6 μg/L) | 0.01 | 0.777 | 0.05 | 0.135 | 0.03 | 0.671 | 0.05 | 0.137 |
|  |  |  |  |  |  |  |  |  |
| Plasma folate (nmol/L) | -0.09 | 0.528 | -0.10 | 0.521 | -0.60 | 0.102 | -0.18 | 0.246 |
| Below reference (6.8 nmol/L) | -0.03 | 0.519 | -0.05 | 0.267 | -0.02 | 0.849 | -0.02 | 0.615 |
|  |  |  |  |  |  |  |  |  |
| Hemoglobin (g/liter) | 0.16 | 0.001 | 0.15 | 0.007 | 1.05 | <0.001 | 0.42 | <0.001 |
| Hemoglobin bel ref. (120) % | -0.08 | 0.005 | -0.07 | 0.017 | -0.31 | <0.001 | -0.11 | <0.001 |
| HCT^4^ | 0.06 | <0.001 | 0.05 | 0.001 | 0.32 | <0.001 | 0.13 | <0.001 |
| MCV^5^ | 0.13 | <0.001 | 0.12 | 0.000 | 0.61 | <0.001 | 0.24 | <0.001 |
| MCH^6^ | 0.03 | 0.002 | 0.03 | 0.020 | 0.21 | <0.001 | 0.09 | <0.001 |
| MCHC^7^ | -0.11 | 0.471 | -0.17 | 0.323 | 0.57 | 0.147 | 0.37 | 0.027 |
|  |  |  |  |  |  |  |  |  |
| **Males** |  |  |  |  |  |  |  |  |
|  |  |  |  |  |  |  |  |  |
| Vitamin D (25OHD_3_ nmol/L) | 0.03 | 0.940 | 0.02 | 0.966 | 1.37 | 0.253 | 0.48 | 0.324 |
| Below reference (50 nmol/L) | 0.01 | 0.911 | -0.09 | 0.289 | 0.10 | 0.608 | -0.03 | 0.719 |
|  |  |  |  |  |  |  |  |  |
| Hemoglobin (g/liter) | 0.08 | 0.132 | 0.16 | 0.027 | 0.67 | <0.001 | 0.34 | <0.001 |
| Hemoglobin bel ref. (130) % | -0.02 | 0.570 | -0.08 | 0.131 | -0.01 | 0.927 | -0.03 | 0.519 |
| HCT^4^ | 0.03 | 0.066 | 0.05 | 0.036 | 0.18 | <0.001 | 0.08 | <0.001 |
| MCV^5^ | 0.08 | <0.001 | 0.10 | <0.001 | 0.36 | <0.001 | 0.17 | <0.001 |
| MCH^6^ | 0.01 | 0.661 | 0.01 | 0.651 | 0.12 | 0.094 | 0.08 | 0.012 |
| MCHC^7^ | 0.00 | 0.987 | 0.08 | 0.392 | 0.17 | 0.394 | 0.14 | 0.110 |
|  |  |  |  |  |  |  |  |  |

**Supplemental table 8**. Association between serum/plasma values of micronutrients and dietary GHGE as quintiles (kg CO_2_eq/day) in the full sample and in different sensitivity analyses excluding participants.

|  | **β-values for quintile regression** | | | | | | | | | |
| --- | --- | --- | --- | --- | --- | --- | --- | --- | --- | --- |
|  | **Vitamin D** | | **Selenium** | | **Zinc** | | **Folate** | | **Hb** | |
|  | **β** | **p** | **β** | **p** | **β** | **p** | **β** | **p** | **β** | **p** |
| **Females** |  |  |  |  |  |  |  |  |  |  |
| *n* | *1332* |  | *1943* |  | *1943* |  | *1477* |  | *15761* |  |
| Full sample | -0.31 | 0.560 | 0.01 | 0.956 | -0.05 | 0.078 | -0.10 | 0.521 | 0.151 | 0.007 |
|  |  |  |  |  |  |  |  |  |  |  |
| *n* | *1000* |  | *1453* |  | *1453* |  | *1116* |  | *11883* |  |
| Excl. recent diet changers | -0.29 | 0.641 | 0.022 | 0.944 | -0.056 | 0.104 | -0.12 | 0.499 | 0.170 | 0.008 |
|  |  |  |  |  |  |  |  |  |  |  |
| *n* | *1047* |  | *1531* |  | *1531* |  | *1187* |  | *12628* |  |
| Excl. potential mis-reporters | -0.796 | 0.212 | -0.186 | 0.561 | -0.537 | 0.112 | -0.206 | 0.269 | 0.285 | <0.001 |
|  |  |  |  |  |  |  |  |  |  |  |
| *n* | *1155* |  | *1693* |  | *1693* |  | *1370* |  | *13426* |  |
| Excl. prevalent cancer | -0.153 | 0.789 | 0.075 | 0.794 | -0.042 | 0.185 | -0.132 | 0.413 | 0.170 | 0.004 |
|  |  |  |  |  |  |  |  |  |  |  |
| *N* | *1322* |  | *1931* |  | *1931* |  | *1461* |  | *15557* |  |
| Excl. prevalent CVD | -0.307 | 0.567 | 0.017 | 0.950 | -0.051 | 0.083 | -0.115 | 0.454 | 0.159 | 0.004 |
|  |  |  |  |  |  |  |  |  |  |  |
| *n* | *1290* |  | *1889* |  | *1889* |  | *1426* |  | *15220* |  |
| Excl. prevalent diabetes | -0.289 | 0.598 | -0.056 | 0.839 | -0.052 | 0.083 | -0.010 | 0.519 | 0.167 | 0.003 |
|  |  |  |  |  |  |  |  |  |  |  |
| *n* | *1110* |  | *1637* |  | *1637* |  | *1309* |  | *12815* |  |
| Excl. prevalent cancer, CVD and DM | -0.075 | 0.899 | 0.049 | 0.866 | -0.046 | 0.152 | -0.151 | 0.361 | 0.191 | 0.002 |
|  |  |  |  |  |  |  |  |  |  |  |
| *N* | *1308* |  | *1908* |  | *1908* |  | *1453* |  | *15547* |  |
| Incl only peri and post-menopausal females | 0.160 | 0.766 | 0.006 | 0.982 | -0.051 | 0.088 | -0.055 | 0.722 | 0.151 | 0.007 |
|  |  |  |  |  |  |  |  |  |  |  |
| *n* | *24* |  | *35* |  | *35* |  | *24* |  | *214* |  |
| Incl only pre-menopausal females | -9.04 | 0.014 | 0.68 | 0.772 | -0.10 | 0.649 | -3.76 | 0.003 | 0.085 | 0.877 |
|  |  |  |  |  |  |  |  |  |  |  |
| **Males** |  |  |  |  |  |  |  |  |  |  |
| *n* | *1570* |  |  |  |  |  |  |  | *10145* |  |
| Full sample | 0.02 | 0.966 |  |  |  |  |  |  | 0.165 | 0.027 |
|  |  |  |  |  |  |  |  |  |  |  |
| *n* | *1235* |  |  |  |  |  |  |  | *7816* |  |
| Excl. recent diet changers | 0.048 | 0.931 |  |  |  |  |  |  | 0.187 | 0.028 |
|  |  |  |  |  |  |  |  |  |  |  |
| *n* | *1376* |  |  |  |  |  |  |  | *8629* |  |
| Excl. potential mis-reporters | 0.331 | 0.535 |  |  |  |  |  |  | 0.223 | 0.008 |
|  |  |  |  |  |  |  |  |  |  |  |
| *n* | *1509* |  |  |  |  |  |  |  | *9684* |  |
| Excl. prevalent cancer | -0.065 | 0.897 |  |  |  |  |  |  | 0.152 | 0.042 |
|  |  |  |  |  |  |  |  |  |  |  |
| *n* | *1494* |  |  |  |  |  |  |  | *9571* |  |
| Excl. prevalent CVD | -0.122 | 0.809 |  |  |  |  |  |  | 0.160 | 0.035 |
|  |  |  |  |  |  |  |  |  |  |  |
| *n* | *1475* |  |  |  |  |  |  |  | *9539* |  |
| Excl. prevalent diabetes | 0.232 | 0.645 |  |  |  |  |  |  | 0.179 | 0.019 |
|  |  |  |  |  |  |  |  |  |  |  |
| *N* | *1357* |  |  |  |  |  |  |  | *8642* |  |
| Excl. prevalent cancer, CVD and DM | -0.044 | 0.933 |  |  |  |  |  |  | 0.149 | 0.057 |
|  |  |  |  |  |  |  |  |  |  |  |
| Model is adjusted for season, age, and storage time for s vitamin D, selenium, zinc and folate. Hb adjusted for season and age. | | | | | | | | | | |

**References:**

1. Nordic Council of Ministers, *Nordic Nutrition Recommendations 2023 - integrating environmental aspects (NNR 2023)*. 2023, Copenhagen: Nordic Council of Minsters.
